# Large scale, robust, and accurate whole transcriptome profiling from clinical formalin-fixed paraffin-embedded samples

**DOI:** 10.1038/s41598-020-74483-1

**Published:** 2020-10-19

**Authors:** Yulia Newton, Andrew J. Sedgewick, Luis Cisneros, Justin Golovato, Mark Johnson, Christopher W. Szeto, Shahrooz Rabizadeh, J. Zachary Sanborn, Stephen Charles Benz, Charles Vaske

**Affiliations:** 1 NantOmics/NantHealth, Inc, 2040 E. Mariposa, El Segundo, CA 90245 USA; 2 ImmunityBio, LLC, 9920 Jefferson Blvd., Culver City, CA 90232 USA

**Keywords:** Biotechnology, Cancer, Molecular biology, Biomarkers, Medical research, Molecular medicine, Computational biology and bioinformatics, Data acquisition, Data integration, Data processing, High-throughput screening, Predictive medicine, Statistical methods, Oncology, Cancer genomics, Cancer screening, Cancer therapy, Tumour biomarkers

## Abstract

Transcriptome profiling can provide information of great value in clinical decision-making, yet RNA from readily available formalin-fixed paraffin-embedded (FFPE) tissue is often too degraded for quality sequencing. To assess the clinical utility of FFPE-derived RNA, we performed ribo-deplete RNA extractions on > 3200 FFPE slide samples; 25 of these had direct FFPE vs. fresh frozen (FF) replicates, 57 were sequenced in 2 different labs, 87 underwent multiple library analyses, and 16 had direct microdissected vs. macrodissected replicates. Poly-A versus ribo-depletion RNA extraction methods were compared using transcriptomes of TCGA cohort and 3116 FFPE samples. Compared to FF, FFPE transcripts coding for nuclear/cytoplasmic proteins involved in DNA packaging, replication, and protein synthesis were detected at lower rates and zinc finger family transcripts were of poorer quality. The greatest difference in extraction methods was in histone transcripts which typically lack poly-A tails. Encouragingly, the overall sequencing success rate was 81%. Exome coverage was highly concordant in direct FFPE and FF replicates, with 98% agreement in coding exon coverage and a median correlation of whole transcriptome profiles of 0.95. We provide strong rationale for clinical use of FFPE-derived RNA based on the robustness, reproducibility, and consistency of whole transcriptome profiling.

## Introduction

Tumor genomic and transcriptomic data typically from targeted panels or microarrays are widely used in clinical cancer diagnosis, prognosis, and therapy recommendations. Numerous studies have shown the utility of RNA sequencing (RNA-Seq) for advancing precision medicine and informing clinical decisions^1–4^. RNA-Seq data is used for gene expression^5^ and allele-specific expression profiling^6^, fusion detection^7^, variant calling^8^ and selection of immunotherapy^9^. However, often only formalin-fixed paraffin-embedded (FFPE) tissues are available for analysis in clinical settings and RNA extracted from such samples can be highly degraded^6,10^. Despite this, FFPE RNA is routinely used for analysis in several FDA-approved tests^11–13^.

To improve data reliability of FFPE-derived RNA, others such as Hoover et al*.* have developed improved methods for extracting high-quality FFPE-RNA by integrating a new micro-homogenizing (mH) tool^14^. In library prep, Zhao et al*.*^15^ demonstrated that compared to mRNA-Seq and microarray, Ribo-Zero-Seq provides equivalent mRNA coverage uniformity, genome-based aligned reads, and high-quality transcript quantification; while having the ability to recover non-polyadenylated and short-transcript RNAs. The ribosomal RNA depletion approach has been widely accepted as the preferred method of RNA isolation for degraded or low-input samples^16–18^. Another improvement comprising use of an “RNA Direct” workflow including targeted capture using the Agilent Strand-Specific RNA Library Prep kit has also been described^19^.

**Figure 1:**
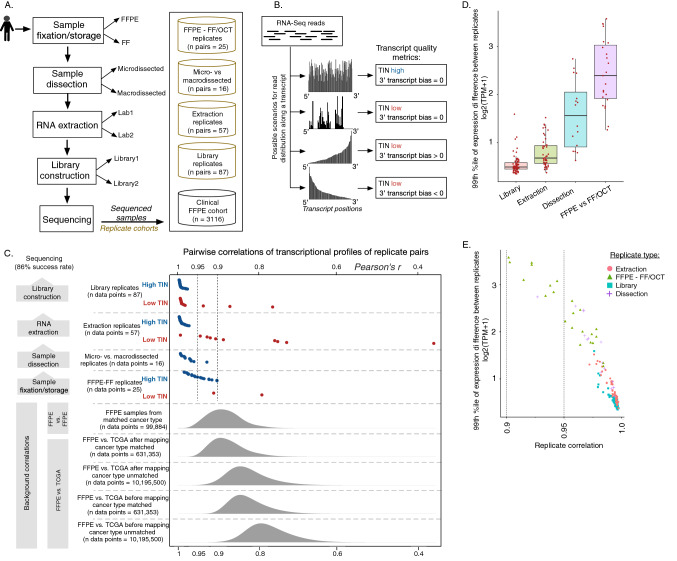
Overview of the study flow scheme, data sets, and sequencing robustness. (**A**) A schematic of the different datasets analyzed in the study is shown. We collected and analyzed replicate sets at each step in the sample collection and sequencing process. (**B**) A schematic representation of post-sequencing transcript quality metrics shows we utilize transcript integrity number (TIN) and 3′ transcript bias metrics that capture the consistency and uniformity of coverage for a given transcript. (**C**) Pairwise correlations of transcriptional profiles of replicate pairs are shown, with each replicate type separated by dashed horizontal lines. The correlations of matched replicates are individual points, broken into high TIN and low TIN groups, based on transcript integrity check as described in Supplementary Methods. Densities on the bottom of the plot indicate various background distributions of correlations. (**D**) Expression difference analysis per each replicate group is presented. The y-axis quantifies the greatest differences in the gene expression quantifications per replicate pair, with types of replicates indicated along the x-axis. This plot shows that the library replicates are the most similar in expression quantification, while FFPE vs. FF/OCT replicates are least similar. (**E**) Expression differences from (**D**) were plotted against correlations of transcriptional profiles in replicate pairs, demonstrating anti-correlation relationships between the two metrics and showing their relationship to the replicate type. For both (**D**) and (**E**), only high-TIN pairs are plotted and only COSMIC cancer genes were used for analysis.

As a contribution to the ongoing improvement in utility of FFPE-RNA, herein we describe our assessment of the reproducibility, quality, and robustness of clinical FFPE-derived RNA at every step of the extraction and sequencing process (Fig. 1A,B, Supplementary Fig. S1) and provide evidence and rationale for the utility of whole transcriptome profiling of FFPE-derived samples (Fig. 1C).

## Results

### Sequencing success and read composition analysis

**Figure 2:**
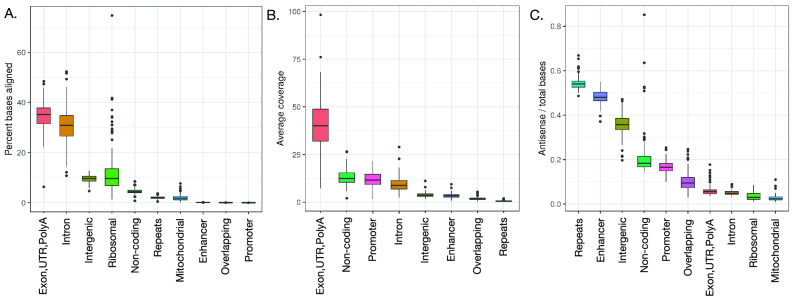
Read composition, coverage, and bias analysis. (**A**) Within the FFPE samples, read composition analysis reveals that most sequenced reads map to exon regions and genomic repeats. (**B**) Exons and ncRNA regions have the most read coverage. Mitochondrial features were not considered for this analysis. (**C**) Read bias within FFPE samples shows repeats regions have the highest percentages of antisense fragments.

In examining a large cohort of FFPE patient samples for RNA sequencing we found that 86% of extracted RNA was successfully prepared for library prep and 94% of those samples had a sufficient amount of non-ribosomal RNA, resulting in an overall RNA sequencing success rate of 81%. The generated FFPE cohort RNA-Seq data resulted in an exonic and intronic mapping rates (Fig. 2A) that are consistent, around 35%, with previously published studies^20,21^ of FFPE-derived RNA-Seq with RNA isolation by rRNA depletion. Exon regions and ncRNAs had the highest coverage (Fig. 2B). When looking at strand bias, we observed that on average 94% of sequenced exomic bases map to a strand concordant with the annotated genomic strand of the mapped gene when no other genes or genomic features were annotated on the other strand. RNA that maps to genomic features in the “Repeats” categories (see Supplementary Methods: Read and base composition analysis) shows higher percentages of anti-sense bases (Fig. 2C), consistent with anti-sense regulation of transcripts. Furthermore, exon bias correlated negatively with TIN (Supplementary Fig. S2A) and positively with GC content (Supplementary Fig. S2B). The strand bias close to 50% for enhancers in Fig. 2C shows that enhancers are transcribed on both strands (the enhancers do not have strand annotations so we refer to the forward strand as “sense” for this analysis). In-depth profiling of stranded RNA coverage in enhancers also supports dual-strand transcription and shows that on average bias switches from the positive strand upstream of enhancers to the negative strand downstream of these sites (Supplementary Fig. S3).

### RNA-Seq replicability

The majority of samples with poor replicability exhibited poor RNA transcript quality when assessed by two separate CLIA-certified labs (Fig. 1C), particularly for the TIN metric^22,23^ (Fig. 1B). Extraction replicates (Supplementary Fig. S4A) with high TIN showed consistent expression quantification with correlation greater than 0.95 (Supplementary Figs. S2B–D, 5A). We found that GC content, fragment size, and exome coverage also affected replicate correlations (Supplementary Fig. 5B–E). When considering other artifacts that might result in differences in assessment between the two labs, we found that there was no bias in template length and exome coverage between them (Supplementary Fig. S6A,B). However, we observed that one of the labs had higher concentration but lower yield and better ribo-depletion quality (Supplementary Fig. S6C,E). The differences in ribo-depletion could account for some of the differences in transcript quality we observed among the extraction replicates (Supplementary Fig. S7). Library replicates showed high reproducibility (Supplementary Fig. S8) and replicates from the same extraction often exhibited high correlations even when corresponding extraction replicate pairs had low correlations (Supplementary Fig. S9), demonstrating sequencing consistency and robustness for the same material. This was also supported by comparison of various quality metrics between corresponding library replicates (Supplementary Figs. S10 and 11). In addition, we did not find that correlations of transcriptional profiles in extraction replicate pairs differed significantly if the genes in the analysis are restricted to COSMIC^24^ cancer genes vs. whole transcriptome (Wilcoxon test *p* = 0.69; Supplementary Fig. S12). We also examined differences between microdissected and macrodissected replicate samples (n pairs = 16) comprising various cancer types (Supplementary Fig. S13A). We found that, while the correlations of transcriptional profiles varied, each replicate sample is most similar to its counterpart (Supplementary Fig. S13B), with the median correlation calculated to be 0.982. In comparisons of gene expression differences in direct replicate pairs, we observed a trend of the most extreme differences increasing from library replicates to microdissection replicates (Fig. 1D). Furthermore, this variance decreases with higher replicate correlation (Fig. 1E). These trends persist whether the whole transcriptome is analyzed or analysis is restricted to COSMIC cancer genes.

### FFPE versus FF/OCT matched samples

In analysis of 25 cases with paired FF/FFPE samples, we found that RNA fragments from FFPE samples tend to be shorter, have higher GC bias (Supplementary Fig. S14A,B), and exhibit lower transcript integrity (Supplementary Fig. S14D,E), which suggested that lower quality RNA is extracted from FFPE. Despite poorer FFPE-derived RNA quality overall, similar transcriptome coverage was observed (Supplementary Fig. S15A). Interestingly, we observed a better mapping rate in FFPE samples (Supplementary Fig. S15B), which could be a direct result of a smaller fragment size in FFPE-derived RNA, as well as higher fraction of reads coming from ribosomal RNA detected in FFPE samples (Supplementary Fig. S15C). Transcriptional profile correlations of FFPE and FF/OCT replicates varied more than extraction replicates, but less than biologically distinct tumors (Fig. 1C; Supplementary Figs. S16 and S17). The observed median correlation of matched pairs was 0.954, much higher than previously reported correlations in expression quantification between FFPE and FF/OCT samples^25–27^. We found that higher correlation between replicate pairs corresponds to pairs with higher TIN, lower GC content, and longer template length, especially in the FFPE cohort (Supplementary Figs. S18 and 19). This is an important finding as it suggests that a sample’s RNA quality, which may easily be quantifiable using several metrics post-sequencing, is crucial to generating clinically-relevant gene expression data. Having well-calibrated RNA quality thresholds ensures robust and reliable gene expression quantification.

**Figure 3:**
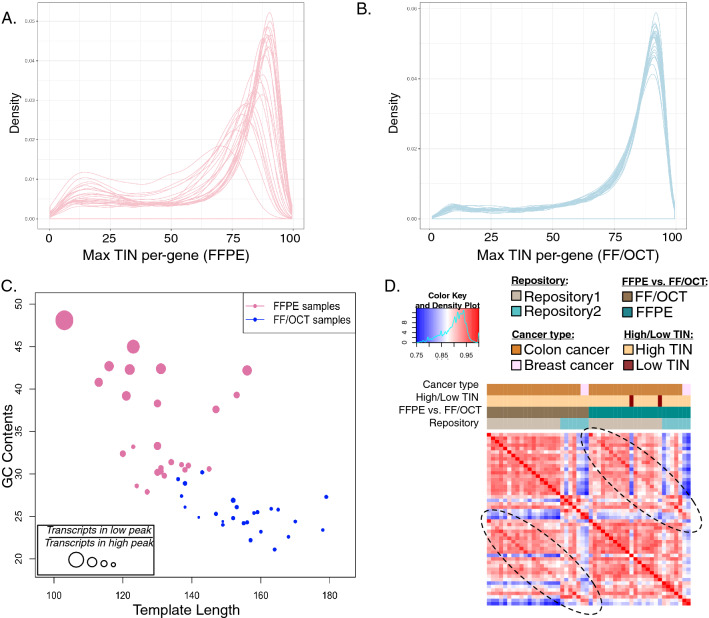
Analysis of FFPE and FF/OCT RNA replicate quality and similarity. Distributions of per-gene TIN in (**A**) FFPE vs. (**B**) FF/OCT replicates are shown. Each line represents a single sample in the cohort. (**C**) Per-sample quality and proportion of transcripts with low TIN are reflected by GC content vs. average template length in FFPE (pink) and FF/OCT (blue) samples. The proportion of genes in the lower peak vs. those in the higher peak in the distributions as shown in (**A**) and (**B**) are indicated by the point size. (**D**) In the correlation heatmap for the combined cohort or replicates, a brighter red color represents greater sample similarity. Samples are ordered the same across both rows and columns. The two peripheral brighter red diagonals indicate these samples are most similar to their FFPE or FF/OCT counterpart than other samples in the same cohort. Sample annotations are described by the color legend.

The consistency of coverage of individual transcripts shows moderate systematic differences between FFPE and FF/OCT replicates. In comparison to their FF/OCT counterparts, some FFPE replicates have many more genes with low TIN (Fig. 3A,B; Supplementary Fig. S20). Per-gene TIN curves in FF/OCT samples consistently follow a smooth unimodal distribution with most genes expressing transcripts with high integrity RNA. Many of the FFPE samples show a bimodal distribution with fewer genes expressing transcripts with high integrity RNA and more genes expressing transcripts with poor quality RNA in comparison to FF/OCT. Shorter average fragment length and higher GC content correlated with transcripts with low TIN values (Fig. 3A–C). Genes encoding zinc finger proteins tended to be amongst low TIN genes in FFPE samples, suggesting that shorter RNA transcripts cause multiple mapping issues for highly homologous genes (*p* = 8.12e−09).

**Figure 4:**
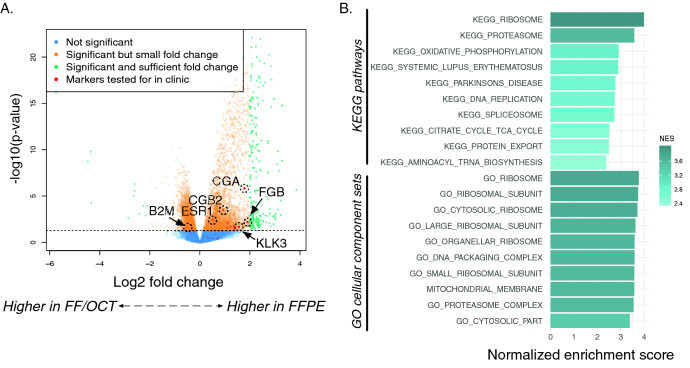
Comparison of transcriptional profiles of FFPE—FF/OCT replicates. (**A**) A volcano plot of results for the linear model fit using weighted least squares for each gene performed on FFPE vs. FF/OCT expression is shown wherein the dashed line indicates an adjusted p-value = 0.05. Green points are genes that are differential for at least a log2 fold change of 2 and are significant after p-value adjustment. Genes for targeted clinical genomic tests (NCI tumor markers list) are shown in red. Several genes have been labeled to provide more insight into which genes are being represented. These labeled genes include those coding for the α subunit of glycoprotein hormones (CGA), beta-2-microglobulin (B2M), chorionic gonadotropin beta-subunit 2 (CGB2), fibrogen B beta chain (FGB), estrogen receptor 1 (ESR1), and kallikrein related peptidase 3 (KLK3). This scatter plot shows that none of these clinically important genes are differential between FFPE and FF/OCT replicates at statistically significant levels. (**B**) GSEA-based pathway enrichments of the differential expression analysis of FFPE vs. FF/OCT cohorts. All pathways listed here are up in FF samples. No statistically significant pathways were found up-regulated in FFPE samples.

We found again that FFPE versus FF/OCT replicate samples are most similar to their corresponding replicate pair (Fig. 3D). Transcriptional profiles between matched samples were highly correlated (Fig. 1C; Supplementary Figs. S16 and 17), with a median correlation of 0.982. Though FFPE vs. FF/OCT replicates exhibited slightly lower correlations than FFPE extraction replicates, the differences are systematic rather than random and thus potentially removable by normalization. Focused analysis of clinically-significant tumor markers^28^ (n = 31) demonstrated similar expression levels in FFPE and FF/OCT samples. In fact, all of the genes were within absolute magnitude of 2 log expression levels (Fig. 4A), indicating consistency of quantification of cancer drivers between FFPE and FF/OCT samples. The expression differences between matched FFPE and FF/OCT samples show enrichment for cellular localization gene sets (Fig. 4B). Transcripts coding for nuclear and cytoplasmic proteins, specifically those involved in DNA packaging, replication, and protein synthesis, were higher in FF/OCT samples in comparison to FFPE samples (linear model fit using weighted least squares for each gene^29^).

### FFPE ribo-depletion vs. FF poly-A selection

**Figure 5:**
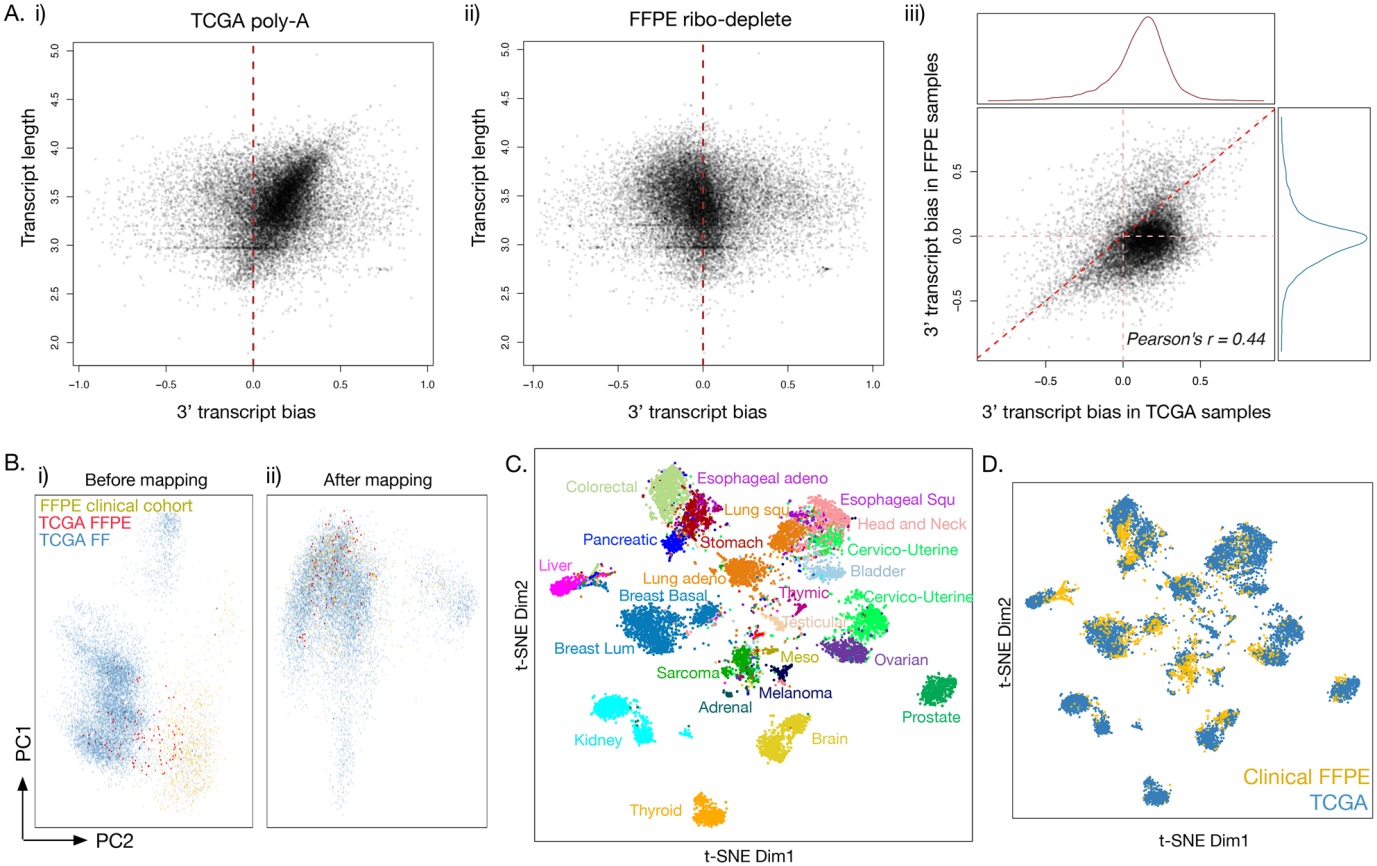
Mapping of the TCGA poly-A cohort to FFPE ribo-deplete samples. Comparison of transcript bias between the two cohorts reveals (**Ai**) strong 3′ bias in TCGA poly-A data (the biggest point density is leaning in the y = x direction, to the right of the x = 0 line) and (**Aii**) slight 5′ bias in FFPE ribo-deplete data (the biggest point density is leaning slightly to the left of the x = 0 line). In parts (**Ai**) and (**Aii**), the dashed red line shows the position on the x-axis where the transcript bias is 0. (**Aiii**) When graphed together, TCGA transcripts have a clear 3′ bias compared to the same transcripts in the FFPE cohort. (**B**) PCA projection of the first 2 principal components of combined FFPE and TCGA cohorts before (i) and after (ii) mapping of TCGA into FFPE RNA-Seq space is represented. (**C**) Shown is a t-SNE projection of the FFPE and TCGA cohorts after the mapping method is applied to TCGA; samples are colored by the cancer type. (**D**) The same t-SNE projection as in (**C**) is shown, but now colored by sample cohort. FFPE samples are yellow, TCGA samples are blue. TCGA and FFPE cohorts mix across cancer type groupings.

To understand systemic differences between FFPE ribo-deplete-extracted RNA and gold standard FF poly-A selected RNA, we compared our large set of clinical FFPE transcriptional profiles (n = 3116), comprising a wide variety of cancer types (Supplementary Fig. S21), and assessed comparability to TCGA FF samples. While 113 of TCGA tumors were ribo-depleted and preserved in FFPE, the majority of TCGA samples (n = 10,379) were poly-A selected and preserved FF. We used the same bioinformatics pipeline, post-sequencing QC, and transcript quantification tools for both datasets. We observed that while TIN is higher in TCGA FF samples (Supplementary Fig. S22A), our FFPE samples show better coverage across most transcripts (Supplementary Fig. S22B). A subset of transcripts in the clinical FFPE cohort exhibited lower TIN than those in TCGA (Supplementary Fig. S22C). This set of transcripts was also found to be enriched for genes coding for zinc finger proteins (*p* = 2.92e−18), consistent with our findings in direct FFPE versus FF replicates. We found that TCGA samples exhibit strong 3′ transcript bias (Fig. 5Ai) for longer transcripts, an expected artifact of poly-A RNA isolation. In contrast, FFPE samples show slight 5′ bias (Fig. 5Aii) and much less dependence on transcript length, suggesting ribo-depletion is better at capturing transcripts with degraded 3′ tails. Accordingly, TCGA transcripts systematically exhibited 3′ bias compared to FFPE transcripts (Fig. 5Aiii).

As a part of the FFPE versus FF TCGA comparison, we developed a computational mapping methodology to project TCGA gene expression data into the same space as clinical FFPE cohort in order to correct for batch effects introduced by combining two such different datasets (Fig. 5Bi–ii). Upon applying this projection, we found the variable driving most RNA-Seq differences was cancer type (Fig. 5C) and not the sample cohort, FFPE or FF (Fig. 5D). When analysis was restricted to COSMIC cancer genes as opposed to the 3000 most varying genes, a projection of clinical FFPE and TCGA FF cohorts into 2-D space showed even less confounding by sample cohort variable, suggesting that not only our mapping methodology is well suited for biomedical oncology research and/or applications of mixed FF and FFPE cohorts, but also that relative expression quantifications of cancer-related genes are highly consistent across FF and FFPE samples. We also found that our mapping methodology performs better in removing platform effects than the commonly used ComBat^30,31^ method, while preserving interpretability of gene-level quantifications (Wilcoxon test *p* <  = 2.2e−16; Supplementary Fig. S23).

**Figure 6:**
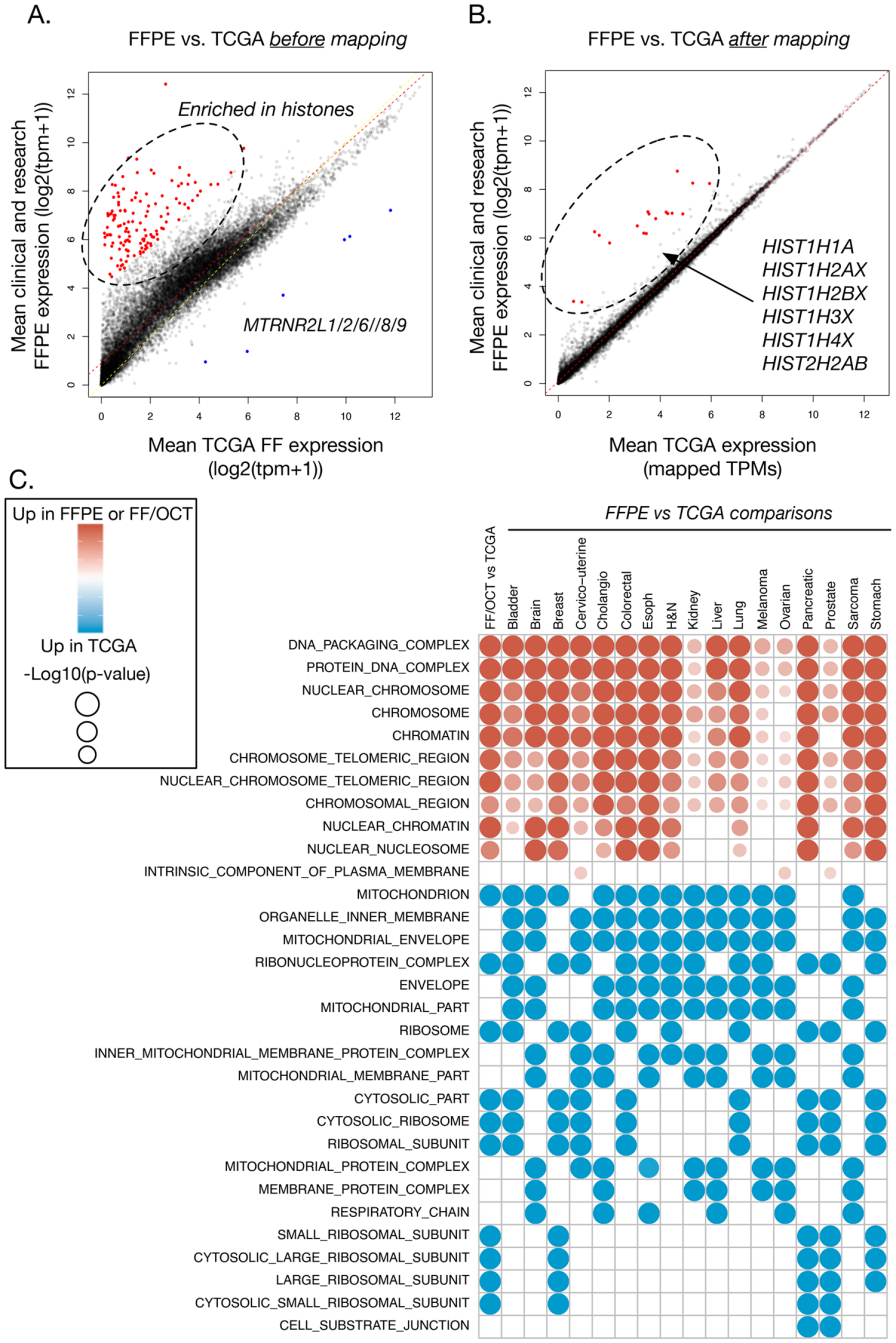
Comparison of transcriptional profiles of the FFPE cohort compared to TCGA samples. Per-gene mean expression comparison between our FFPE and TCGA cohorts before (**A**) and after (**B**) the mapping of TCGA into FFPE RNA-Seq space is shown. (**C**) Pathway MSigDB enrichments for each of the cancer type, as well as FF/OCT samples, vs. TCGA of matched cancer type were computed using top differential genes; enrichment strength is based on FWER p-value.

Prior to mapping TCGA RNA-Seq onto our clinical FFPE cohort, we compared expression levels of TCGA FFPE (n = 113) and TCGA FF (n = 10,266) samples to the clinical FFPE cohort separately. We found that TCGA FFPE expression is more consistent with clinical FFPE samples, with few highly differential outliers (Supplementary Fig. S24). The majority of genes downregulated in the clinical FFPE samples in comparison to TCGA FFPE cohort encode for mitochondrial proteins (Fig. 6A, Supplementary Fig. S24B), which is expected given the use of different library preparation methodologies: RNAs coding for mitochondrial proteins have high sequence similarity to ribosomal RNA and are therefore often removed by ribo-depleting oligos. Since it is important to understand the prognostic and diagnostic utility of FFPE-derived RNA biomarkers, we assessed such utility of several well-known molecular markers in breast carcinoma samples. We found that FFPE breast cohort stratification, based on expression quantifications of these markers, is consistent with TCGA FF breast samples (Supplementary Fig. S25), suggesting that FFPE-derived RNA markers retain their value for clinical assay use.

The differences between clinical FFPE and TCGA FF cohorts are compounded by differing RNA isolation methodologies. The genes that were most upregulated in the FFPE cohort include various histones (Fig. 6B), which are often not poly-adenylated^32,33^ and therefore are not captured by poly-A selection. This difference was observed before (Fig. 6A) and after (Fig. 6B) our mapping methodology was applied, highlighting that differences in RNA capture methods have a strong effect on downstream differential gene expression analysis. GSEA of tissue-specific differential expression (Supplementary Fig. S26) showed enrichment for nuclear cellular activity gene sets in FFPE samples and enrichment of cytoplasmic activity gene sets, such as mitochondrial organization and ribosomal functions, in TCGA samples (Fig. 6C).

## Discussion

RNA-Seq data from FFPE-derived RNA has shown sufficient precision and sensitivity for biomarker discovery in breast cancer^34^ and has even been utilized for analysis of microRNA (miRNA) as a tumor classifier/target for therapeutic intervention^35^. But the use of RNA-Seq in clinical settings remains infrequent because of uncertainty concerning the quality of FFPE-derived RNA.

While others have addressed the issue of FFPE and FF comparison in smaller studies^36,37^ and have investigated which protocols are best for preparation of RNA libraries^20^ or tools for read alignment on FFPE-derived samples^38,39^, our study concentrated on examining any intrinsic biases in the FFPE-derived gene expression data and assessing its utility in clinical applications. Our study also contains the largest, in number of samples, FFPE cohort published to date. We performed comprehensive analysis of various types of replicates at every stage of RNA sample fixation/storage, extraction, and sequencing and show that—when quality is controlled—gene expression quantification of FFPE-derived RNA is robust, consistent and reproducible. We introduce methodology for evaluating sample RNA quality post-sequencing, and show that when this method is applied to samples, it produces high quality, high confidence results in replicate datasets.

We identified systematic trends and technical artifacts that affect gene expression profiling in FFPE samples. For example, we demonstrate that the sample fixation method accounts for the apparent increased expression of nuclear and cytoplasmic proteins in FF/OCT samples and the apparent increased expression of transmembrane and secreted proteins in FFPE samples. Similarly, some of the transcripts detected at higher rates in FF/OCT samples are genes that encode for histone proteins, which have been shown to undergo critical chemical modifications in FFPE samples^40^. Further, while FF-derived RNA transcripts with a poly-A tails may be deemed more clinically relevant because they are destined for translation, some non-polyadenylated transcripts have been shown to be translated into peptides^41^ and are important in tumorigenesis or biomarkers in cancer^42–44^.

The results presented here comport with and represent a significant expansion on previous findings. Jovanovic et al*.*^45^ demonstrated that subtyping accuracy of FFPE and FF triple negative breast cancer (TNBC) samples increased with sequencing depth, and that samples with shorter archiving time (< 4 years) were subtyped more accurately, presumably due to reduced RNA degradation which is consistent with others investigators^46^. However, Jovanovic focused their analysis on genes that did not vary between platform type, tissue processing, and RNA isolation techniques, tacitly suggesting that certain transcripts were unreliable and should be excluded. Like Jovanovic, we noticed an inverse relationship between the archiving time and RNA quality of the samples. Here, we present evidence that focusing on driver genes does not increase quality substantially, but rather that quality metrics such as TIN and TB can be used to exclude poor quality samples and transcripts post-sequencing. Our study suggests a quality threshold cutoff for TIN metric that can be used to identify samples with RNA quality too poor to perform reliable genomic analysis. Additionally, we provide a computational methodology to normalize out systemic differences in detected ribosomal RNA content in sequenced samples resulting from the differences in degree of sample degradation as well as differences in rRNA depletion among different FFPE samples.

Fortifying confidence in transcriptomic analysis of FFPE-derived RNA in clinical settings opens up a broad spectrum of possibilities to advance patient care and may lead to RNA sequencing becoming indispensable as a diagnostic and prognostic technology that may also reveal new targets for therapeutic intervention.

## Methods

### Patients samples, microdissection and preparation

Tumor samples were obtained from patients across multiple clinical centers. These samples were analyzed as part of routine clinical care when the oncologists ordered a molecular diagnostic assay from NantOmics/NantHealth, Inc. These samples comprised a wide variety of solid tumor types (Supplementary Fig. S21). All methods were carried out in accordance with relevant guidelines and regulations and in the Declaration of Helsinki; informed consent was obtained from all subjects or, if subjects were under 18, from a parent and/or legal guardian; data were analyzed under approved company policies for handling de-identified patient information. This study underwent ethics review and was approved by an internal ethical review board at NantOmics/NantHealth, Inc.

From each FFPE tissue block, a single 4 µM and three 10 µM tissue sections were cut. The initial 4 μM section was subjected to hematoxylin and eosin (H&E) staining and reviewed by a board-certified pathologist who identified and marked regions of malignancy. Using the marked-up image as a guide, areas of tissue were macrodissected to collect the desired regions.

Laser microdissection of FFPE sections was performed on a subset of FFPE samples for comparison of direct microdissection vs. macrodissected replicates (n pairs = 16). The pathologist marked up an H&E image, then mirrored that mark-up onto the FFPE section to guide the laser microdissection.

### In-house FFPE RNA extraction and sequencing

The RNeasy FFPE Kit (Qiagen, Venlo, Netherlands) was used for extraction and extracted material quantified using a Qubit fluorometer (ThermoFisher Scientific, Waltham, MA, USA). Samples with total RNA quantities of greater than 600 ng were prepped in duplicate using a KAPA Stranded RNA-Seq Kit with RiboErase (Kapa Biosystems, Wilmington, MA, USA) and sequenced to a target depth of 200 M reads on the Illumina HiSeq platform (Illumina, San Diego, CA, USA). Duplicate preps were both sequenced if there was enough material to do separate technical library preparations for each, otherwise 2× material from a single library prep was loaded to reach 200 M reads.

### Computational pipeline for generation of sequencing data

Bowtie^47^, RSEM, and custom software were used for alignment and transcript quantification. STAR^48^ alignments were performed on a subset of clinical FFPE samples (n = 114). RefSeq^49^ (build 73) on hg19 was used to define transcripts for each pipeline. See methods for read and base composition analysis in Supplementary Methods. A subset of the data are available upon request (email corresponding author).

### The Cancer Genome Atlas (TCGA) data

We downloaded TCGA RNA-Seq FASTQ files from the GDC Legacy Data Archive (https://portal.gdc.cancer.gov/legacy-archive/) and applied the same bioinformatics pipeline for transcript quantification to these data as for the FFPE clinical cases. Data from our processing of TCGA data are available upon request (email corresponding author).

### Transcript Integrity Number (TIN)

TIN was used to computationally assess RNA quality post-sequencing^22^. Descriptions of the derivation of TIN score and TIN-derived quality metrics are described in detail in Supplementary Methods.

### TIN-derived quality metrics

We derived a per-sample quality score by taking the median of non-zero TINs within a single sample. A sample is defined as high quality if it had a TIN greater than 50. Specifically, for replicate pairs, the pair is high quality if both samples in the pair pass the TIN cutoff.

Additionally, we computed a per-transcript TIN score by averaging the number of reads per base within the transcript, as well as a per-gene TIN score by taking the median non-zero TIN score within each sample. Per-gene TIN was determined by taking the maximum TIN of the canonical transcript for that gene.

### Transcript 3′ bias

While TIN scoring is useful for analyzing non-uniform coverage, it is not sufficient to detect particular end biases that result from degradation. Here we introduce an additional measure of transcript uniformity—the Transcript Bias (TB) score—to specifically address 5′–3′ coverage bias. This measure is based on the difference between the observed distribution of reads and a uniform distribution with the same average coverage. Details of TB score calculation can be found in Supplementary Methods.

### Differential gene expression using linear model fit

To compute differential gene expression we utilized the LIMMA^50^ R package to perform linear model fit using weighted least squares for each gene.

### Gene expression divergence in replicate pairs analysis

To quantify expression levels differences for each replicate pair and gene, we compared the average expression vs. expression difference plotted as Bland–Altman plots (Supplementary Fig. S27 for extraction replicates; Supplementary Fig. S28 for microdissected replicates; Supplementary Figs. S29 and S30 for FFPE vs. FF/OCT replicates) for the following sets of genes: whole transcriptome, COSMIC cancer genes, and, in some cases, genes targeted by clinical genomic tests (NCI tumor markers list). Variance cannot be defined using two replicates, thus we introduce the concept of per-gene divergence as the absolute value of the difference in replicate pair expression and computed 99th %tile of divergence within the sample.

### Correlation of RNA expression replicates

We sought to calculate the correlation of expression profiles in replicates. To ensure the variance in RNA detection limits did not bias these analyses, we focused on genes with at least moderate levels of expression by filtering out genes with low expression using a cutoff of mean rescaled TPM > 1. This left us with expression profiles of 15,992 genes to calculate the Pearson correlation using log2 transformed rescaled TPMs with an offset of 1 TPM.

### Pathway enrichment analysis for FFPE vs. FF/OCT replicates

We analyzed the function of genes appearing as most differentially expressed between FFPE vs. FF/OCT by first identifying significantly differential genes in paired t-tests, then using Gene Set Enrichment Analysis (GSEA) queried against KEGG and GO cellular component MSigDB^51^ gene sets. The top 10 enriched pathways for each gene set were studied further for functional significance.

### Pathway enrichment analysis for FFPE vs. TCGA and FF/OCT vs. TCGA

We sought to functionally annotate genes that are differentially detected between the library preps used in FFPE vs. TCGA. First, we limited analysis to tissues with at least n = 30 samples in both datasets (Supplemental Fig. S26) to ensure that tissue-specific expression did not bias analysis. Next, we extracted the top 150 most-differentially expressed genes (up- or down-regulated) by t-test. Next, we applied a hypergeometric test using the MSigDB pathway online tool with GO cellular component gene sets to find pathways that were enriched for said genes.

### Other methods

Transcript Integrity Number (TIN) score and TB score; enrichment analysis of genes with low transcript integrity in FFPE vs. FF/OCT replicates and FFPE vs. TCGA cohorts; normalization to overcome variations in rRNA depletion quality across the FFPE cohort; exomic feature annotations and other genomic features considered; features coverage and read and base composition analysis; analysis of enhancers; per-transcript and per-HUGO-gene expression quantification from RNA-seq data; mapping of TCGA data into the FFPR RNA-seq space are described in Supplementary Methods.

## Supplementary information

Supplementary Information 1.

Supplementary Legends.

Supplementary Table 1.

Supplementary Information 2.

Supplementary Figure 1.

Supplementary Figure 2.

Supplementary Figure 3.

Supplementary Figure 4.

Supplementary Figure 5.

Supplementary Figure 6.

Supplementary Figure 7.

Supplementary Figure 8.

Supplementary Figure 9.

Supplementary Figure 10.

Supplementary Figure 11.

Supplementary Figure 12.

Supplementary Figure 13.

Supplementary Figure 14.

Supplementary Figure 15.

Supplementary Figure 16.

Supplementary Figure 17.

Supplementary Figure 18.

Supplementary Figure 19.

Supplementary Figure 20.

Supplementary Figure 21.

Supplementary Figure 22.

Supplementary Figure 23.

Supplementary Figure 24.

Supplementary Figure 25.

Supplementary Figure 26.

Supplementary Figure 27.

Supplementary Figure 28.

Supplementary Figure 29.

Supplementary Figure 30.

Supplementary Figure 31.

Supplementary Figure 32.

Supplementary Figure 33.

## Abbreviations

FF: Fresh frozen
FFPE: Formalin-fixed paraffin-embedded
TCGA: The Cancer Genome Atlas
RNA-Seq: RNA-sequencing
TPM: Transcript per million
TIN: Transcript integrity number
TB: Transcript bias
OCT: Optimal cutting temperature
NC: Non-coding
NMD: Nonsense mediated decay
miRNA: MicroRNA
poly-A: Poly-adenylated
FDA: Food and Drug Administration
