# Supplementary material for: Large scale, robust, and accurate whole transcriptome profiling from clinical formalin-fixed paraffin-embedded samples
Source: Sci Rep. 2020 Oct 19;10:17597. doi: 10.1038/s41598-020-74483-1 (PMC7572424; doi:10.1038/s41598-020-74483-1)
Supplement: Supplementary file 1 — Supplementary Information 1. [file 41598_2020_74483_MOESM1_ESM.docx]

**Large scale, robust, and accurate whole transcriptome profiling from clinical formalin-fixed paraffin-embedded samples**

Yulia Newton^1^*, Andrew J. Sedgewick^1^, Luis Cisneros^1^, Justin Golovato^2^, Mark Johnson^1^, Christopher W. Szeto^1^, Shahrooz Rabizadeh^2^, J. Zachary Sanborn^1^, Stephen Charles Benz^1^, Charles Vaske^1^

*Corresponding Author

^1^NantOmics/NantHealth, Inc, 2040 E. Mariposa, El Segundo, CA 90245, USA

^2^ImmunityBio, LLC 9920 Jefferson Blvd., Culver City, CA 90232, USA

Yulia Newton: ynewton@nanthealth.com

Luis Cisneros: cisluis@asu.edu

Justin Golovato: Justin.golovato@immunitybio.com

Mark Johnson: Mark.Johnson@nantomics.com

Christopher Szeto: cszeto@nanthealth.com

Andrew J. Sedgewick: asedgewick@nanthealth.com

Shahrooz Rabizadeh: [Sr@immunitybio.com](mailto:Shahrooz@immunitybio.com)

J. Zachary Sanborn: zsanborn@nanthealth.com

Stephen Charles Benz: steve.benz@nanthealth.com

Charles Vaske: cvaske@gmail.com

**Running Title**

Transcriptome profiling from FFPE

**Key words**

Formalin-fixed paraffin-embedded (FFPE) tissue, RNA quality, ribo-deplete, transcriptome profiling, RNA-Seq

**List of abbreviations**

Fresh frozen (FF), formalin-fixed paraffin-embedded (FFPE), The Cancer Genome Atlas (TCGA), RNA-Sequencing (RNA-Seq), transcript per million (TPM), transcript integrity number (TIN), transcript bias (TB), optimal cutting temperature (OCT), non-coding (NC), nonsense mediated decay (NMD), microRNA (miRNA), poly-adenylated (poly-A), Food and Drug Administration (FDA)

**Human genes**

The α subunit of glycoprotein hormones (CGA), beta-2-microglobulin (B2M), chorionic gonadotropin beta-subunit 2 (CGB2), fibrogen B beta chain (FGB), estrogen receptor 1 (ESR1), and kallikrein related peptidase 3 (KLK3).

**Full name, mailing address, phone and fax numbers, and email address of the corresponding author:** Yulia Newton, NantHealth, Inc, 2040 E. Mariposa, El Segundo, CA 90245 USA 1.855.WHY.NANT (1.855.949.6268), YNewton@nanthealth.com

**Supplemental Methods**

***Transcript Integrity Number (TIN) score***

TIN is a convenient measure introduced by Wang *et al* ^1^ to computationally assess RNA quality post-sequencing by assuming that systematic *in vitro* degradation of a transcript results in regions with shallow read depths. Others have suggested application of TIN to determine quality of RNA-seq data. ^2^ The TIN score captures the uniformity of coverage in an array of reads per location. Given an RNA transcript of N nucleotides with a read coverage per base n_i (i=1…N), the relative coverage is defined as:

$$P_{i}=\frac{n_{i}}{\sum_{d=1}^{N} n_{i}}$$

From this definition, the coverage uniformity across the transcript can be quantified by the cumulative sum of a monotonically decreasing function of $P_{i}$. The Shannon entropy $H=-\sum_{d=1}^{N} P_{i}*log(P_{i})$ method is a good fit for this type of problem. In this way, following the definition in Wang *et al.*, the TIN score is defined as:

$$TIN=(\frac{100}{N})*e^{H}$$

It is important to point out that the sum *H* is maximized when the coverage is perfectly uniform (i.e. $P_{i}=\frac{1}{N}$), and nucleotide positions that have no reads ($n_{i}=0$) do not contribute to it. As such, having uneven coverage (e.g. due to 5’ degradation), or having many nucleotides with no read coverage would result in low TIN scores.

Unlike in Wang *et al*., we analyzed TIN scores from all coding positions instead of subsampling every 100 bases (which was performed to reduce computational complexity). This comprehensive TIN scoring was performed to guarantee the best possible quality.

***TB score***

The Transcript Bias (TB) score takes into account the normalized cumulative sum of the read coverage as a function of the location:

$$s_{i}=\frac{\sum_{j<i} n_{j}}{\sum_{k=1} n_{k}}$$

If reads are uniformly distributed along the transcript, this cumulative sum will increase linearly with procession in locus from 5’ to 3’ in the integration process because $n_{i}\propto i\Rightarrow s_{i}\propto\frac{i}{N}$. If there is 5’ bias in coverage, then $s_{i}$ will show a greater-than-linear increase and saturate at an intermediate location. Conversely, if there is 3’ bias then the $s_{i}$ will increase sub-linearly and then increase sharply at an intermediate locus. Thus, by subtracting the cumulative sum from that of a linear model, we can quantify transcript-end bias by means of the integral:

$$S=\int s_{i}\delta x-\frac{1}{2}$$

Here we implement this integral as the trapezoid Riemann area under the discrete curve, $s_{i}$, with $x=\frac{i}{N}$ being the normalized location along the transcript. The term $\frac{1}{2}$ corresponds to the area under the uniform model, $y=x$, thus $S$ measures the difference between the observed coverage profile and the null hypothesis (Supplementary Fig S31).
The sum $S$ is positive if the original distribution of reads is 5’ biased and negative if it is 3’ biased. Therefore, we can define the TB score as:

$$TB=-2*S$$

which is normalized such that $TB\to-1$ when transcript reads are biased to the 5’ end, and $TB\to+1$ when biased to the 3’ end. Even though uniform coverage distribution would yield a $TB\sim0$, the converse is not necessarily true; a measure of $TB=0$ indicates a more general condition of symmetry between the transcript 5’ and 3’ halves.

***Enrichment analysis of genes with low transcript integrity in FFPE vs. FF/OCT replicates and FFPE vs. TCGA cohorts***

To analyze genes with consistently low TIN in FFPE samples compared to FF/OCT (Supplementary Fig S20) or TCGA samples (Supplementary Fig S22C), we used a threshold of TIN = 40 and determined in how many samples a given gene appears below or above this threshold. Fisher’s Exact Test was used to compute a p-value, applied Bonferroni for p-value correction, and selected all genes with an adjusted p-value <= 0.05. We observed that many of these genes encode zinc fingers. Therefore, we obtained the list of zinc fingers from HGNC web site and computed the hypergeometric test p-value for enrichment of zinc fingers in the set of statistically significant genes from the Fisher Exact Test.

For FFPE versus TCGA analysis we filtered out transcripts with average coverage of fewer than 3 reads per-base. We selected the top 2,000 transcripts for which the median per-transcript TIN was most differential between the two cohorts and saw these were also enriched for zinc fingers. We then computed a hypergeometric test p-value against the list of zinc finger genes obtained and described earlier in FFPE vs. FF/OCT replicates per-gene TIN analysis.

***Normalization to overcome variations rRNA depletion quality across the FFPE cohort***

Captured expression levels of RNA45S5 (45S Pre-Ribosomal 5), a non-coding gene product, can be used as a proxy for the quality of ribosomal RNA depletion. Across the cohort of 2,713 FFPE samples, we found a large variation in the ratio of expected counts of RNA45S5 to all expected counts in a given sample (Supplementary Fig S32A), and the variation was independent of other biological variables such as tissue of origin (Supplementary Fig S32B). This variance is indicative of high variance in the quality of ribosomal RNA depletion. Because RSEM calculates TPM based on all transcripts in each sample including RNA45S5, the variability in ribosomal depletion will affect TPM values for other genes. We observed that samples with high levels of RNA45S5 contamination had reduced transcriptional similarity to biologically similar tumors (Supplementary Fig S32C, “Before rescaling”) which prompted us to develop a single-sample normalization method to combat this variance in ribo-deplete sample quality and apply it to all samples reported here (Supplementary Fig S32C, “After rescaling”). We rescaled the TPM values of each sample so that the TPMs for all protein coding genes (genes with at least one isoform beginning with NM_ in RefSeq) in the sample sum up to 1 million. This step allows for a more uniform and interpretable comparison of expression levels across samples.

***Genomic features***

Given a list of genomic features, one can count the amount of reads that map to each. To calculate statistics for the coverage fraction associated with each feature, counting must take feature overlaps into account to avoid double counting reads. To allow this, we used HTSeq ^3^ a Python package that provides infrastructure to process data from high-throughput sequencing assays. When counting read coverage in regions that align to more than one feature, we partition these regions into sub-regions (or steps) that have unique labels. Thus, if for instance features A and B overlap, the partitions will correspond to steps {A}, {A, B} and {B} for each section of uniquely defined feature annotation. The output of this process is a table of non-overlapping genomic regions uniquely annotated by the feature or list of features.

***Exomic feature annotations***

The locations for basic exomic features use the positions annotated in RefSeq. Exons and UTRs were identified for coding transcripts. Exons in transcripts that are non-coding but that are associated with coding genes (i.e. genes associated with coding RNA transcripts) were designated as non-sense mediated decay (nmd) transcripts. Exons in non-coding transcripts that are not associated with coding genes (thus only associated with pseudogenes) were deemed Non-Coding (nc) transcripts. Introns and intergenic regions were identified as the appropriate flanking regions around these features.

***Other genomic features considered***

1. From the UCSC Genome Browser - RepeatMasker (rmsk) table:

a. Long Interspersed Nuclear Elements (LINE)

b. Alu short interspersed nuclear elements non-autonomous retrotransposons (ALU)

c. Non-Alu short interspersed nuclear elements non-autonomous retrotransposons (SINE)

2. From the UCSC Genome Browser:

a. Human Body Map large intergenic non-coding RNAs and transcripts of uncertain coding potential (lincRNA)

b. Precursor forms of microRNAs (miRNA)

c. mRNA PolyAdenlyation Sites (polyA)

3. From the FANTOM5 Atlas:

a. Human permissive enhancers (enhancer)

4. From the The Eukaryotic Promoter Database (EDP):

a. Promoter Regions (promoter)

***Features Coverage***

Read coverage for all annotated regions was calculated by counting the number of bases mapped to the sense strands in each region and dividing by the length of the region. Because our region annotations do not overlap there is no risk of double counting bases in this step. To obtain genomic feature bias we computed the ratio of bases mapped to antisense strand over total bases for each feature. In order to accurately profile strand bias, sense and antisense coverages were separately calculated for features that had no annotation other than “intergenic” on the opposite strand. Intergenic and enhancer regions do not have strand annotations so for coverage calculations bases mapping to both strands are counted, but to assess strand bias the forward strand is set as “sense” for these regions.

***Read and base composition analysis***

We then grouped the genomic elements by their annotations into the following categories:

- *Exon, UTR, PolyA*: elements with any combination of annotations for exons, 5’ and 3’ UTRs and PolyA tails for protein coding genes (including noncoding isoforms of coding genes).
- *Ribosomal*: elements annotated as rRNA that may overlap with annotations for intergenic, lincRNA or other noncoding RNA.
- *Enhancer:* elements annotated as enhancers; may overlap with intron or intergenic annotations.
- *Promoter*: elements annotated as promoters; may overlap with intron or intergenic annotations.
- *Repeats*: elements annotated as Alu elements, Long Interspersed Nuclear Elements (LINE), Short interspersed nuclear elements (SINE), and Long Terminal Repeats Retrotransposons (LTR); these may overlap with intron or intergenic annotations.
- *Non-coding*: elements annotated as the lincRNA, miRNA, and noncoding pseudogenes.
- *Mitochondrial*: elements only annotated as mitochondrial genes.
- *Intergenic*: elements only annotated as intergenic.
- *Intron*: elements only annotated as introns.

The “Exon, UTR, PolyA*”*, “Intron” and “Ribosomal” categories were allowed to overlap with “Non-coding”, “Enhancer”, “Promoter” and “Repeat” annotations, but not each other. The “Non-coding” category was allowed to overlap with “Enhancer”, “Promoter” and “Repeat” regions. The remaining categories were not allowed to overlap with any others. Regions with disallowed overlaps were annotated as “Overlapping”. Base composition analysis was performed by computing percent of total bases that aligned to each of these categories.

***Analysis of Enhancers***

For analysis of enhancer features, we considered regions annotated as enhancer features corresponding to either full enhancer regions or sections of enhancer regions that do not intersect with any genomic feature other than intergenic regions. Using the coordinates defining these regions, we use SAMTools ^4^ to extract the read profiles in each enhancer plus 2,000 bases flanking both their 3' and the 5' ends. Only regions at least 80 bases long were considered, as very short ones are likely to be small leftover sections of longer enhancers that intersect other features and are not really very informative. The read coverage for each of these features was calculated for each sample and fractions of total read coverage estimated. Following Chen *et al* ^5^ for each enhancer region the stranded read profile as a function of the distance to the center of the region was calculated. An example of a centered profile in chromosome 4 (start:189048639; end:189048958) for a single sample is shown in Supplementary Fig S3 (part A), where the reads per base in both the positive (sense) and the negative (antisense) strands (plus total) is shown. Additionally, a bias coefficient function c was calculated (Supplementary Fig S3B) as the difference between positive and negative strand reads relative to the total number of reads as c = (pos - neg)/tot, in such a way that for a given base a value of c = 1 means that all reads in that base are from the positive strand, while c = -1 means that all reads are negative strand and c = 0 means that there is equal number of positive and negative strand reads.

Furthermore, in order to characterize the typical behavior across all enhancer regions in a given sample, we calculated the mean centered profile per base. In this methodology, for each enhancer region the coding regions that intersect the 2kb flanks are subtracted, so that we only consider the part of the read profile that does not overlap with exons. Then, for each possible base location the average of the number of stranded reads for all the enhancer intervals that have data at that particular distance from the reference center was estimated. This produces a normalized average profile in which coding regions are subtracted to avoid biasing due to gene expression, and hence is a good representation of the typical expression profile of eRNAs alone in the sample (Supplementary Fig 3C-D).

***Per-transcript and per-HUGO-gene expression quantification from RNA sequencing data***

Transcript quantification was computed as an average number of reads per base within each transcript. In order to assess gene expression, we obtained transcript per million (TPM) from RSEM output of RNA sequencing data. To compute per-sample gene expression profiles we summarized per-gene expression as a sum of TPM for all isoforms of a given gene.

***Projecting TCGA data into the FFPE RNA-Seq space***

We obtained public RNA sequencing data for 10,528 poly-A capture samples from the TCGA project for 33 different cancer. In order to transform these samples to a similar space to our reference FFPE samples, we utilized a quantile normalization ^6^ procedure, with FFPE samples’ expression quantiles as the target distribution (Supplementary Fig S33). We performed this quantile normalization procedure for each gene/feature. We also excluded zero expression values from both source and target datasets (per-gene) and attached those after the mapping was completed. In order to account for differences in cancer type composition between the two datasets, we implemented this quantile normalization in two steps as follows: 1. The TCGA dataset was divided into two subsets: in the first, cancer type composition was matched (number of samples in each cancer type comprises at least the same percent of total samples as in the target FFPE dataset); and in the second, with the remaining samples that were not included into the first subset. 2. The first subset was quantile normalized as described above. The second subset was normalized using quantiles of the first subset as a source. The results generated by this method were compared to those from the ComBat ^7,8^ method and we observed that our mapping method performed better than ComBat while preserving interpretable gene-level quantifications (see next section for description of the test to compare the two methods).

***Comparison of ComBat method to our projection method to map TCGA data into FFPE space***

We compared the two mapping methods by comparing how much the two datasets mix after the methods are applied by measuring the amount of scatter, or clumpiness, of datasets in a 2-D t-SNE projection of the high dimensional RNA-Seq data. For each point in Supplementary Fig S23B (for ComBat method) and Fig 5D (for our projection method), we computed Euclidean distance in 2-D space between that point and the top 5 nearest points in the other dataset (in TCGA dataset for FFPE samples and in the clinical FFPE dataset for TCGA samples). We computed median distance among each set of 5 neighbors (Supplementary Fig S23C) and computed statistical significance of the difference between the two density curves using Wilcoxon test.

***Assessing utility of prognostic breast cancer molecular markers***

In order to assess the prognostic power of well-known clinical diagnostic and prognostic biomarkers in our study FFPE cohort as compared to TCGA FF samples, we compared expression levels of ESR1 (PR), PGR (PR), and ERBB2 (HER2) genes and previously published PAM50 molecular subtypes in the TCGA breast cohort (Supplementary Fig S25Ai). As expected, we found that expression levels of these markers track with the molecular subtypes. We found that the distributions of expression levels of these markers in the FFPE breast samples is consistent with TCGA (Supplementary Fig S25Aii) and exhibit similar expression correlation patterns between each other as in TCGA cohort (Supplementary Fig S25B), suggesting that these markers have the same prognostic utility in the FFPE-derived RNA as in the FF-derived RNA.

**Supplementary References**

1 Wang, L. *et al.* Measure transcript integrity using RNA-seq data. *BMC Bioinformatics* **17**, 58, doi:10.1186/s12859-016-0922-z (2016).

2 Son, K., Yu, S., Shin, W., Han, K. & Kang, K. A Simple Guideline to Assess the Characteristics of RNA-Seq Data. *Biomed Res Int* **2018**, 2906292, doi:10.1155/2018/2906292 (2018).

3 Anders, S., Pyl, P. T. & Huber, W. HTSeq--a Python framework to work with high-throughput sequencing data. *Bioinformatics* **31**, 166-169, doi:10.1093/bioinformatics/btu638 (2015).

4 Li, H. *et al.* The Sequence Alignment/Map format and SAMtools. *Bioinformatics* **25**, 2078-2079, doi:10.1093/bioinformatics/btp352 (2009).

5 Chen, H. *et al.* A Pan-Cancer Analysis of Enhancer Expression in Nearly 9000 Patient Samples. *Cell* **173**, 386-399.e312, doi:10.1016/j.cell.2018.03.027 (2018).

6 Bullard, J. H., Purdom, E., Hansen, K. D. & Dudoit, S. Evaluation of statistical methods for normalization and differential expression in mRNA-Seq experiments. *BMC Bioinformatics* **11**, 94, doi:10.1186/1471-2105-11-94 (2010).

7 Muller, C. *et al.* Removing Batch Effects from Longitudinal Gene Expression - Quantile Normalization Plus ComBat as Best Approach for Microarray Transcriptome Data. *PloS one* **11**, e0156594, doi:10.1371/journal.pone.0156594 (2016).

8 Johnson, W. E., Li, C. & Rabinovic, A. Adjusting batch effects in microarray expression data using empirical Bayes methods. *Biostatistics* **8**, 118-127, doi:10.1093/biostatistics/kxj037 (2007).
