# Supplementary material for: Large scale, robust, and accurate whole transcriptome profiling from clinical formalin-fixed paraffin-embedded samples
Source: Sci Rep. 2020 Oct 19;10:17597. doi: 10.1038/s41598-020-74483-1 (PMC7572424; doi:10.1038/s41598-020-74483-1)
Supplement: Supplementary file 3 — Supplementary Table 1. [file 41598_2020_74483_MOESM3_ESM.docx]

Supplementary Figure1: Overview of different analyses performed by this study and how they fit into different stages of RNA sequencing process.

Supplementary Figure 2: Exon bias vs. TIN (A) and GC contents (B). Exon bias correlates negatively with TIN and positively with GC contents.

Supplementary Figure 3: Enhancer RNA analysis. A) An example of stranded coverage for a single enhancer. X-axis shows +/- 2kb of the start of the enhancer start site. Blue line shows that coverage of the negative strand and uptake in coverage at the start of enhancer site. B) Bias coefficient plot for the same enhancer as in part A. Bias coefficient describes the fraction of all reads being from positive strand at a given position. C) Stranded coverage summarized across all enhancers of length of at least 80 bases. Green line shows average reads on the negative strand at a given site and shows an increase in negative strandedness at the enhancers’ sites. D) Bias coefficient aggregated across all enhancers of length of at least 80 bases.

Supplementary Figure 4: Analysis of extraction replicates. A) Distribution of cancer types within extraction replicates cohort. B) Distribution of correlations between extraction replicates by tumor type. C) Correlation heatmap of extraction replicates sorted by cancer type. Both rows and columns are ordered by the same sample order. D) Extraction replicates ordered by their correlations. Yellow line indicates .99, pink line indicates .95, and red line indicates .9 level.

Supplementary Figure 5: Comparison of replicate correlations and various sample quality metrics in direct extraction replicates. For each replicate pair we show a given quality metric for both replicates, on the y-axis we show correlation between these pairs; each replicate pair is connected by a dashed line. A) GC contents. B) Average exome coverage. C) Median per-sample TIN. D) Average template length. E) rRNA depletion quality.

Supplementary Figure 6: Comparison of sample quality metrics in extraction replicates. A) Average template length comparison shows similar results for both labs. B) Average exome coverage is highly correlated between two labs. C-D) Lab2 had higher concentration but lower yield. E) rRNA depletion was better at Lab2.

Supplementary Figure 7: Comparison of TIN in extraction replicates. A) Median per-sample TIN is slightly higher in Lab2. B) Median per-sample TIN tends to be higher in Lab2 samples. C) Median per-transcript TIN is highly correlated between two labs.

Supplementary Figure 8: Comparison of per-sample median TIN in direct library replicates, showing high reproducibility in sample quality of the same material.

Supplementary Figure 9: Correlations of transcriptional profiles of extraction replicates vs. library replicates. It shows that even when one of the extraction replicates has low quality we can still get highly correlated library replicates from that sample.

Supplementary Figure 10: Comparison of various sample quality metrics in direct library replicates, showing high concordance in these metrics between paired samples. A) Average template length. B) GC contents. C) Average exome coverage. D) rRNA depletion quality.

Supplementary Figure 11: Comparison of replicate correlations and various sample quality metrics in direct library replicates. In A - E, for each replicate pair we show a given quality metric for both replicates, on the y-axis we show correlation between these pairs; each replicate pair is connected by a dashed line. A) Average template length. B) GC contents. C) Average exome coverage. D) rRNA depletion quality. E) Median per-sample TIN. F) Comparison of per-transcript median TIN between library replicate pairs, showing high consistency in transcript quality.

Supplementary Figure 12: Correlations of transcriptional profiles of FFPE - FF/OCT replicates with different gene sets (whole transcriptome on the y-axis vs. COSMIC cancer genes on the x-axis). Correlations in pairs where at least one sample has low TIN are indicated in red. Wilcoxon Test statistical test that examines if these correlations form a different distribution results in p-value = 0.69, indicating that these distributions are likely the same.

Supplementary Figure 13: Analysis of microdissected vs. macrodissected replicates. A) Replicate correlations and their tumor types by sample. B) Heatmap of replicate correlations. Samples in both columns and rows are ordered in the same order. Bright red colors indicate higher correlations. Two off-center diagonals of bright red indicate that microdissected samples are most similar to their macrodissected counterparts.

Supplementary Figure 14: Comparison of sample quality metrics in FFPE vs. FF/OCT replicates. A) Average template length comparison shows higher values in FF/OCT samples. B) GC bias comparison shows that FFPE samples have higher GC contents. C) Per-sample median TIN in FF/OCT (x-axis) vs. FFPE (y-axis) replicates plot shows that TIN tends to be higher in FF/OCT samples. D) Replicate correlation vs. quality (TIN) difference between replicate pairs shows inverse relationship between these two metrics. E) Per-transcript quality comparison shows high correlation in transcript quality with a subset of transcripts having higher TIN in FF/OCT samples.

Supplementary Figure 15: Comparison of coverage and read mapping in FFPE vs. FF/OCT replicates. A) Average transcript coverage is highly consistent between replicates. B) Percent reads mapped (mapped reads / total reads) in FFPE vs. FF/OCT replicates. The box plot shows higher mapping rate in FFPE samples. C) Comparison of ribosomal RNA fraction in FFPE and FF/OCT replicate samples. FFPE samples tend to have higher ribosomal RNA content than corresponding FF/OCT samples.

Supplementary Figure 16: Comparison of per-gene expression levels across the whole transcriptome in log2(rescaled TPM + 1) space of FFPE vs. FF/OCT replicates. All pairs in dataset 1 are colorectal tumor type. Pairs 1-5 in dataset 2 are colon tumor type, while pairs 6 and 7 are breast tumor type. Red line indicates y = x slope. Yellow line indicates linear regression line.

Supplementary Figure 17: Comparison of per-gene expression levels across COSMIC cancer genes in log2(rescaled TPM + 1) space of FFPE vs. FF/OCT replicates. All pairs in dataset 1 are colorectal tumor type. Pairs 1-5 in dataset 2 are colon tumor type, while pairs 6 and 7 are breast tumor type. Red line indicates y = x slope. Yellow line indicates linear regression line.

Supplementary Figure 18: Comparison of replicate correlations and various sample quality metrics in FFPE vs. FF/OCT replicates. For each replicate pair we show a given quality metric for both replicates, on the y-axis we show correlation between these pairs; each replicate pair is connected by a dashed line. A) Average template length. B) GC contents. C) Average exome coverage. D) Median per-sample TIN. E) rRNA depletion quality.

Supplementary Figure 19: Relationships between different quality metrics in FFPE vs. FF/OCT replicates. Data repositories are indicated by the point shape and tissue/storage type is indicated by the point color.

Supplementary Figure 20: Per-gene RNA transcript integrity comparison between FFPE and FF/OCT replicates. In each histogram we plot distribution of maximum Transcript Integrity Numbers (TIN) across all transcripts that represent a gene symbol. On the left of each pair is the FFPE sample and on the right is the FF/OCT sample. Pearson correlation shown under FFPE sample labels is the correlation of transcriptional expression profiles between the samples in each pair.

Supplementary Figure 21: Distribution of cancer types within this study’s FFPE cohort. The counts are listed by the sample type, separated by comma (clinical, extraction replicates, microdissection replicates, FFPE-FF/OCT replicates). Only those samples with cancer type annotations available are used in these counts. Excluded from this plot are 1,485 unlabeled and research samples.

Supplementary Figure 22: Analysis of various sample quality metrics in our study's FFPE cohort vs. TCGA samples. A) Median per-sample TIN as density plots. B) Per-transcript coverage scatterplot shows that our study has better coverage for most transcripts. C) Per-transcript median TIN shows that, while most transcripts show concordance in quality, a subset of transcripts have higher quality in TCGA. These transcripts are significantly enriched in those coding for zinc finger genes.

Supplementary Figure 23: Comparison of mapping of TCGA RNA-Seq data into clinical FFPE using ComBat method vs. the method proposed by our study. A) t-SNE projection of the combined data, before mapping TCGA into clinical FFPE, showing which dataset each sample belongs to. B) t-SNE projection of the combined data, after mapping TCGA into clinical FFPE, showing which dataset each sample belongs to. C) Comparison of distance between clinical FFPE and TCGA datasets after TCGA dataset is mapped/projected into clinical FFPE dataset using ComBat method and using our proposed method (see Supplemental Methods). We measured the amount of “scatter”, or clumpiness, of the two datasets by computing, for each point in part B of this figure as well as part D of Figure 5, the Euclidean distance between that point and top 5 nearest points in the 2-D space from the other dataset. For each point we computed the median distance between these neighbors. The density plots represent the median distances. The dashed lines indicate median values of each distribution. This plot shows that our method produces more “mixing” (smaller distance) between the datasets.

Supplementary Figure 24: Comparison of per-gene mean expression between this study's FFPE cohort and TCGA samples before mapping of TCGA into FFPE RNA-Seq. A) Comparison against TCGA FF samples. B) Comparison against TCGA FFPE samples.

Supplementary Figure 25: Predictive value of ER, PR, and HER2 in clinical diagnostics of breast cancer samples, as compared between the TCGA FF and clinical FFPE cohorts. A) Principal component analysis (PCA) of TCGA (i) and FFPE (ii) breast samples with samples colored by expression levels of ER, PR, and HER2, with brighter color indicating higher expression. For TCGA cohort we include a PCA plot with samples colored by their published PAM50 subtypes. B) Correlation matrices of individual biomarkers within the FFPE (i) and TCGA (ii) cohorts.

Supplementary Figure 26: Overview of differential expression contrasts performed in this study for Figure 6C.

Supplementary Figure 27: Expression comparison of extraction replicates. There are too many samples for a single figure, so in the interest of space we show two examples of highly (on the left) and poorly (on the right) correlated replicates. Two columns show representative pairs with high (left) and low (right) correlations. A) Correlation plots using different gene sets: whole transcriptome (top) and COSMIC cancer genes (bottom). B) Bland–Altman plots using different gene sets: whole transcriptome (top) and COSMIC cancer genes (bottom).

Supplementary Figure 28: Bland–Altman plots using whole transcriptome (on the left for each replicate pair) and COSMIC cancer genes (on the right for each replicate pair) for microdissected vs. macrodissected replicates.

Supplementary Figure 29: Bland–Altman plots using whole transcriptome in log2(rescaled TPM + 1) space of FFPE vs. FF/OCT replicates. All pairs in dataset 1 are colorectal tumor type. Pairs 1-5 in dataset 2 are colon tumor type, while pairs 6 and 7 are breast tumor type. Red line indicates y = x slope. Yellow line indicates linear regression line.

Supplementary Figure 30: Bland–Altman plots using COSMIC cancer genes in log2(rescaled TPM + 1) space of FFPE vs. FF/OCT replicates. All pairs in dataset 1 are colorectal tumor type. Pairs 1-5 in dataset 2 are colon tumor type, while pairs 6 and 7 are breast tumor type. Red line indicates y = x slope. Yellow line indicates linear regression line.

Supplementary Figure 31: Graphical explanation cumulative sums used for the TB score. In the top panel a uniform coverage produces a linear cumulative distribution s_i and thus the integral S is close to zero. In the case of a read coverage biased to the left end of the transcript (middle panel) the cumulative function s_i grows faster than linearly producing a positive value of S and if the coverage is biased to the right end of the transcript (lower panel) then s_i would grow slower than linearly and S have a negative value.

Supplementary Figure 32: Analysis of ribo-depletion quality across the clinical and research FFPE cohort. A) Fraction of RNA45S5 expected counts was computed for each sample as a proxy for amount of ribosomal RNA remaining in the sample after ribo-deplete process. Distribution of these fractions is presented as a histogram. B) RNA45S5 fraction per each cancer type. C) Two breast cancer samples are presented. The first sample has the lowest fraction of RNA45S5 among all breast tumor samples (RNA45S5 low). The second sample has the highest fraction of RNA45S5 among all breast tumor samples (RNA45S5 high). We show that applying per-sample rescaling helps reduce variability driven by technical artifacts and make expression levels more interpretable across biologically similar samples.

Supplementary Figure 33: Schematic diagram for the method of mapping external datasets into the FFPE cohort for joint analysis of RNA-Seq data.
