# Supplementary material for: Large scale, robust, and accurate whole transcriptome profiling from clinical formalin-fixed paraffin-embedded samples
Source: Sci Rep. 2020 Oct 19;10:17597. doi: 10.1038/s41598-020-74483-1 (PMC7572424; doi:10.1038/s41598-020-74483-1)
Supplement: Supplementary file 4 — Supplementary Information 2. [file 41598_2020_74483_MOESM4_ESM.docx]

SUPPLEMENTARY DATA INDEX

1. **Supplemental Tables (SupplementalTable1.xlsx)**
   1. Library replicates
   2. Microdissected vs. whole slide replicates
   3. Extraction replicates
   4. FFPE-FF/OCT replicates
   5. FFPE vs FF/OCT pathway enrichments
   6. Library replicates per-transcript TIN
   7. Microdissected vs whole slide replicates per-transcript TIN
   8. Extraction replicates per-transcript TIN
   9. FFPE-vs-FF/OCT replicates per-transcript TIN
   10. Clinical and research FFPE cohort per-transcript TIN
   11. Clinical and research FFPE cohort per-sample TIN
   12. Outlier genes in FFPE-vs-FF/OCT replicate comparison
   13. Outlier genes in FFPE-vs-FF/OCT replicate comparison
   14. Differential expression for all contrasts
   15. Outlier genes in FFPE-vs-TCGA comparison
   16. FFPE-vs-FF/OCT replicates per-gene TIN analysis results
   17. GSEA pathway enrichments in FFPE or FF/OCT when compared to TCGA
   18. MSigDB pathway enrichments upregulated in FFPE or FF/OCT when compared to TCGA
   19. MSigDB pathway enrichments downregulated in FFPE or FF/OCT when compared to TCGA
   20. Direct comparison of replicate pair expression values
2. **Supplemental Figures**
   1. Supplemental Figure 1
   2. Supplemental Figure 2
   3. Supplemental Figure 3
   4. Supplemental Figure 4
   5. Supplemental Figure 5
   6. Supplemental Figure 6
   7. Supplemental Figure 7
   8. Supplemental Figure 8
   9. Supplemental Figure 9
   10. Supplemental Figure 10
   11. Supplemental Figure 11
   12. Supplemental Figure 12
   13. Supplemental Figure 13
   14. Supplemental Figure 14
   15. Supplemental Figure 15
   16. Supplemental Figure 16
   17. Supplemental Figure 17
   18. Supplemental Figure 18
   19. Supplemental Figure 19
   20. Supplemental Figure 20
   21. Supplemental Figure 21
   22. Supplemental Figure 22
   23. Supplemental Figure 23
   24. Supplemental Figure 24
   25. Supplemental Figure 25
   26. Supplemental Figure 26
   27. Supplemental Figure 27
   28. Supplemental Figure 28
   29. Supplemental Figure 29
   30. Supplemental Figure 30
   31. Supplemental Figure 31
   32. Supplemental Figure 32
   33. Supplemental Figure 33
3. **Supplemental figure legends in a word document (supplemental_figure_legends.docx)**
4. **Supplementary Methods**
   1. Transcript Integrity Number (TIN) score
   2. TB score
   3. Enrichment analysis of genes with low transcript integrity in FFPE vs. FF/OCT replicates and FFPE vs. TCGA cohorts
   4. Normalization to overcome variations rRNA depletion quality across the FFPE cohort
   5. Genomic features
   6. Exomic feature annotations
   7. Other genomic features considered
   8. Features Coverage
   9. Read and base composition analysis
   10. Analysis of Enhancers
   11. Per-transcript and per-HUGO-gene expression quantification from RNA sequencing data
   12. Projecting TCGA data into the FFPE RNA-Seq space
   13. Comparison of ComBat method to our projection method to map TCGA data into FFPE space
   14. Assessing utility of prognostic breast cancer molecular markers
