# Supplementary material for: Large scale, robust, and accurate whole transcriptome profiling from clinical formalin-fixed paraffin-embedded samples
Source: Sci Rep. 2020 Oct 19;10:17597. doi: 10.1038/s41598-020-74483-1 (PMC7572424; doi:10.1038/s41598-020-74483-1)
Supplement: Supplementary file 6 — Supplementary Figure 2. [file 41598_2020_74483_MOESM6_ESM.pdf]

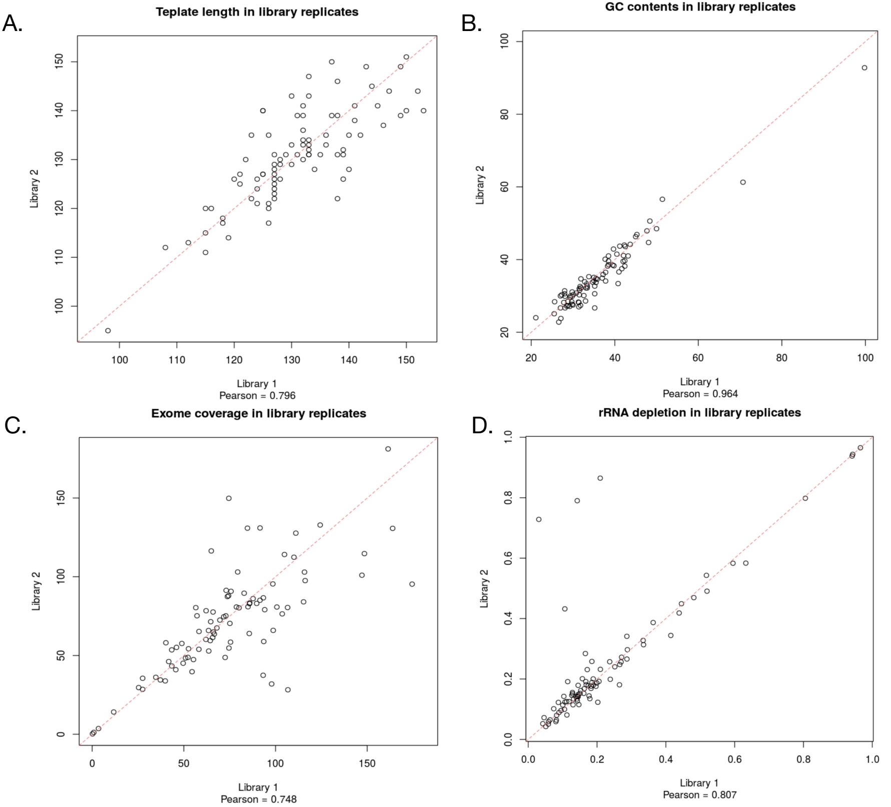

Supplementary Figure 10: Comparison of various sample quality metrics in direct library replicates, showing high concordance in these metrics between paired samples. A) Average template length. B) GC contents. C) Average exome coverage. D) rRNA depletion quality.
