# Supplementary material for: Large scale, robust, and accurate whole transcriptome profiling from clinical formalin-fixed paraffin-embedded samples
Source: Sci Rep. 2020 Oct 19;10:17597. doi: 10.1038/s41598-020-74483-1 (PMC7572424; doi:10.1038/s41598-020-74483-1)
Supplement: Supplementary file 8 — Supplementary Figure 4. [file 41598_2020_74483_MOESM8_ESM.pdf]

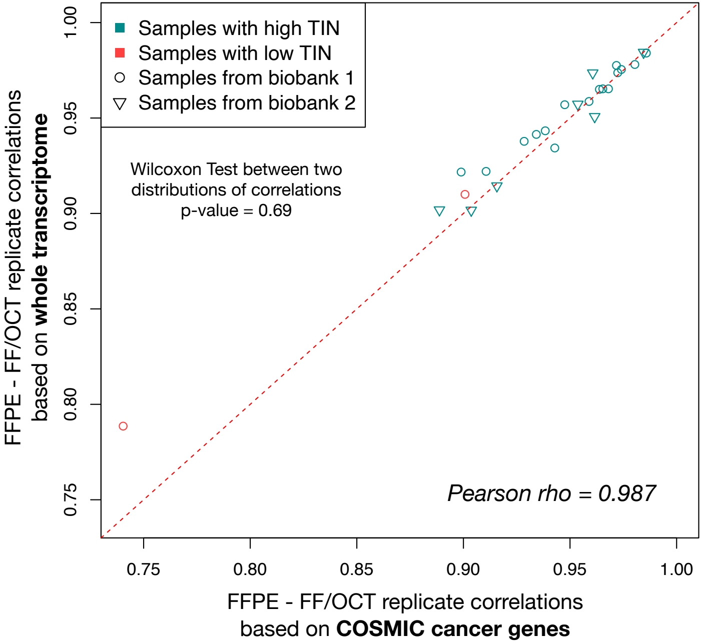

Supplementary Figure 12: Correlations of transcriptional profiles of FFPE - FF/OCT replicates with different gene sets (whole transcriptome on the y-axis vs. COSMIC cancer genes on the x-axis). Correlations in pairs where at least one sample has low TIN are indicated in red. Wilcoxon Test statistical test that examines if these correlations form a different distribution results in p-value = 0.69, indicating that these distributions are likely the same.
