# Supplementary material for: Large scale, robust, and accurate whole transcriptome profiling from clinical formalin-fixed paraffin-embedded samples
Source: Sci Rep. 2020 Oct 19;10:17597. doi: 10.1038/s41598-020-74483-1 (PMC7572424; doi:10.1038/s41598-020-74483-1)
Supplement: Supplementary file 9 — Supplementary Figure 5. [file 41598_2020_74483_MOESM9_ESM.pdf]

A. Distribution of cancer types in microdissected vs. macrodissed sample pairs and transcriptional profile correlations

Sample	Cancer type	Microdis vs wholeslide correlation
Sample1	Bone and Soft Tissue Cancers (including Sarcoma); mesenchymal chondrosarcoma	0.993
Sample2	Head and Neck; poorly diff cancer of parotid gland	0.960
Sample3	Colon	0.985
Sample4	Breast	0.970
Sample5	Brain; anaplastic astrocytoma	0.991
Sample6	Kidney; renal cell carcinoma	0.982
Sample7		0.927
Sample8	Ovarian	0.971
Sample9	Brain	0.994
Sample10	Breast	0.992
Sample11	Soft Tissue Sarcoma	0.993
Sample12	Prostate	0.983
Sample13	Ovarian	0.967
Sample14	Brain; salivary duct carcinoma	0.967
Sample15	Sarcomatoid carcinoma	0.982
Sample16	Breast	0.968

B. Transcriptome correlation heat map in microdissected vs. macrodissected cohorts

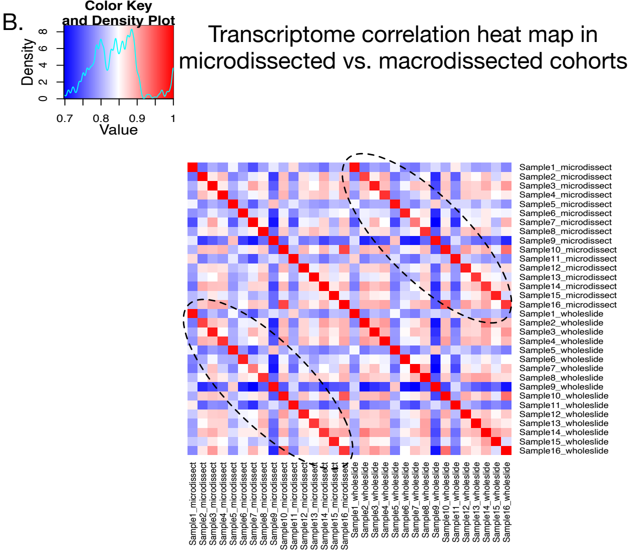

Supplementary Figure 13: Analysis of microdissected vs. macrodissected replicates. A) Replicate correlations and their tumor types by sample. B) Heatmap of replicate correlations. Samples in both columns and rows are ordered in the same order. Bright red colors indicate higher correlations. Two off-center diagonals of bright red indicate that microdissected samples are most similar to their macrodissected counterparts.
