# Supplementary material for: Large scale, robust, and accurate whole transcriptome profiling from clinical formalin-fixed paraffin-embedded samples
Source: Sci Rep. 2020 Oct 19;10:17597. doi: 10.1038/s41598-020-74483-1 (PMC7572424; doi:10.1038/s41598-020-74483-1)
Supplement: Supplementary file 10 — Supplementary Figure 6. [file 41598_2020_74483_MOESM10_ESM.pdf]

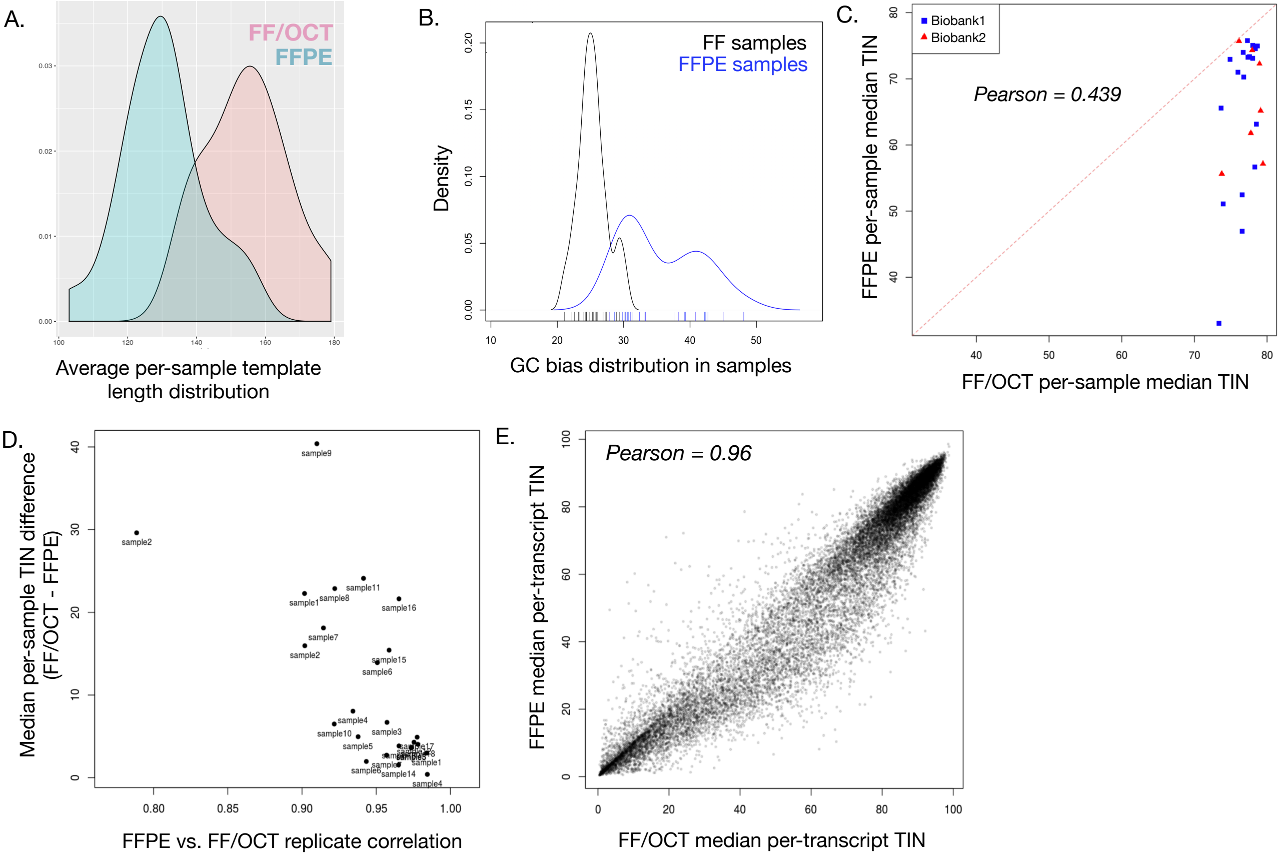

Supplementary Figure 14: Comparison of sample quality metrics in FFPE vs. FF/OCT replicates. A) Average template length comparison shows higher values in FF/OCT samples. B) GC bias comparison shows that FFPE samples have higher GC contents. C) Per-sample median TIN in FF/OCT (x-axis) vs. FFPE (y-axis) replicates plot shows that TIN tends to be higher in FF/OCT samples. D) Replicate correlation vs. quality (TIN) difference between replicate pairs shows inverse relationship between these two metrics. E) Per-transcript quality comparison shows high correlation in transcript quality with a subset of transcripts having higher TIN in FF/OCT samples.
