# Supplementary material for: Large scale, robust, and accurate whole transcriptome profiling from clinical formalin-fixed paraffin-embedded samples
Source: Sci Rep. 2020 Oct 19;10:17597. doi: 10.1038/s41598-020-74483-1 (PMC7572424; doi:10.1038/s41598-020-74483-1)
Supplement: Supplementary file 11 — Supplementary Figure 7. [file 41598_2020_74483_MOESM11_ESM.pdf]

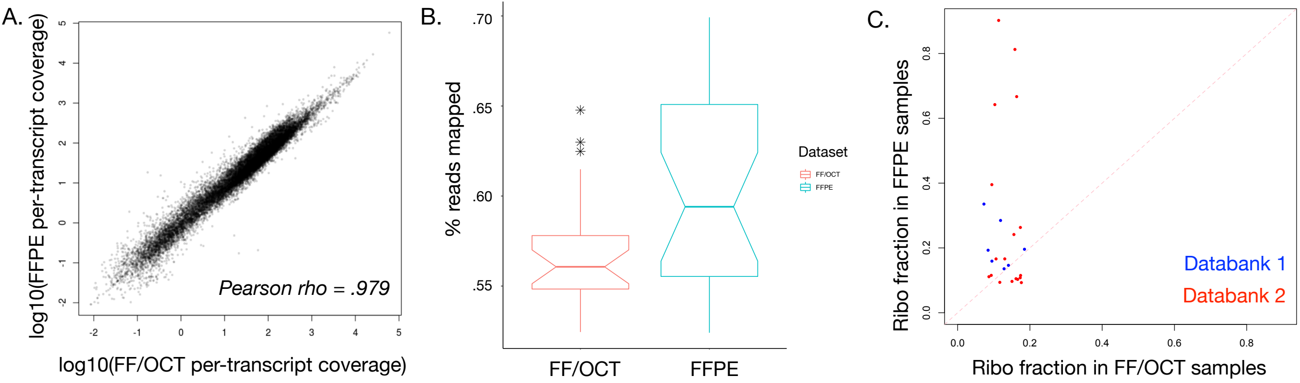

Supplementary Figure 15: Comparison of coverage and read mapping in FFPE vs. FF/OCT replicates. A) Average transcript coverage is highly consistent between replicates. B) Percent reads mapped (mapped reads / total reads) in FFPE vs. FF/OCT replicates. The box plot shows higher mapping rate in FFPE samples. C) Comparison of ribosomal RNA fraction in FFPE and FF/OCT replicate samples. FFPE samples tend to have higher ribosomal RNA content than corresponding FF/OCT samples.
