# Supplementary material for: Large scale, robust, and accurate whole transcriptome profiling from clinical formalin-fixed paraffin-embedded samples
Source: Sci Rep. 2020 Oct 19;10:17597. doi: 10.1038/s41598-020-74483-1 (PMC7572424; doi:10.1038/s41598-020-74483-1)
Supplement: Supplementary file 14 — Supplementary Figure 10. [file 41598_2020_74483_MOESM14_ESM.pdf]

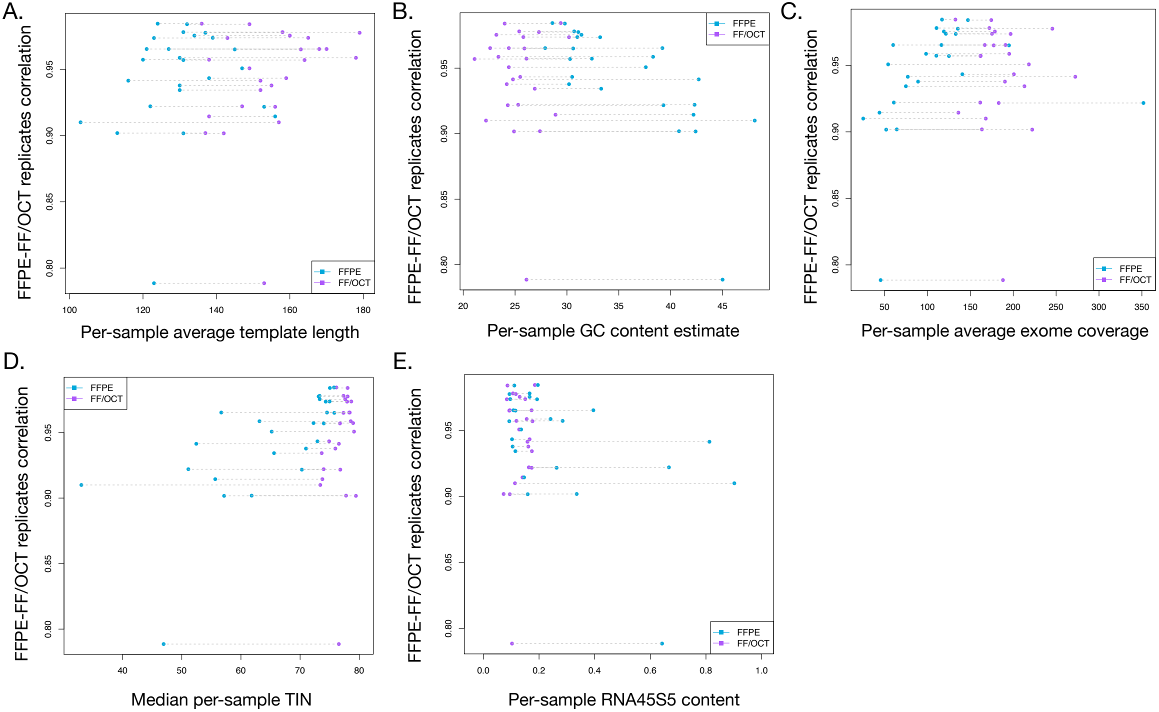

Supplemental Figure 18: Comparison of replicate correlations and various sample quality metrics in FFPE vs. FF/OCT replicates. For each replicate pair we show a given quality metric for both replicates, on the y-axis we show correlation between these pairs; each replicate pair is connected by a dashed line. A) Average template length. B) GC contents. C) Average exome coverage. D) Median per-sample TIN. E) rRNA depletion quality.
