# Supplementary material for: Large scale, robust, and accurate whole transcriptome profiling from clinical formalin-fixed paraffin-embedded samples
Source: Sci Rep. 2020 Oct 19;10:17597. doi: 10.1038/s41598-020-74483-1 (PMC7572424; doi:10.1038/s41598-020-74483-1)
Supplement: Supplementary file 15 — Supplementary Figure 11. [file 41598_2020_74483_MOESM15_ESM.pdf]

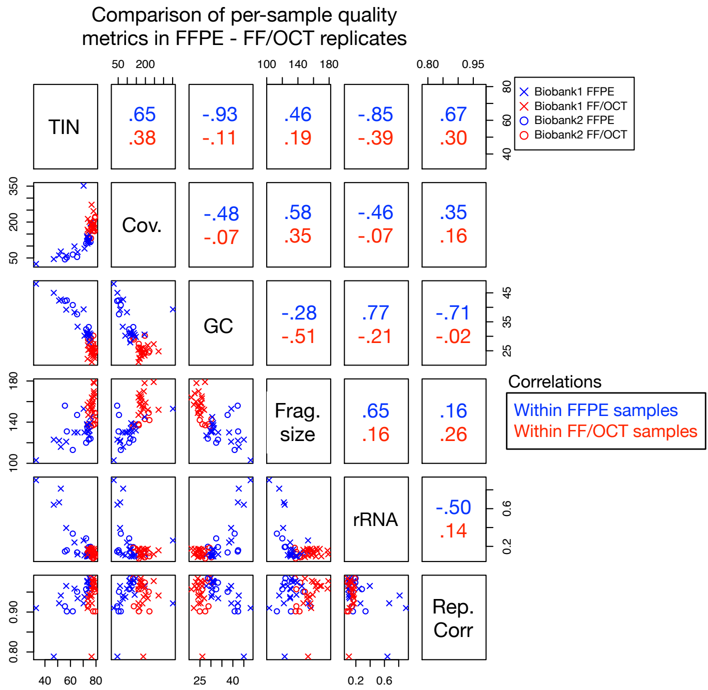

Supplementary Figure 19: Relationships between different quality metrics in FFPE vs. FF/OCT replicates. Data repositories are indicated by the point shape and tissue/storage type is indicated by the point color.
