# Supplementary material for: Large scale, robust, and accurate whole transcriptome profiling from clinical formalin-fixed paraffin-embedded samples
Source: Sci Rep. 2020 Oct 19;10:17597. doi: 10.1038/s41598-020-74483-1 (PMC7572424; doi:10.1038/s41598-020-74483-1)
Supplement: Supplementary file 16 — Supplementary Figure 12. [file 41598_2020_74483_MOESM16_ESM.pdf]

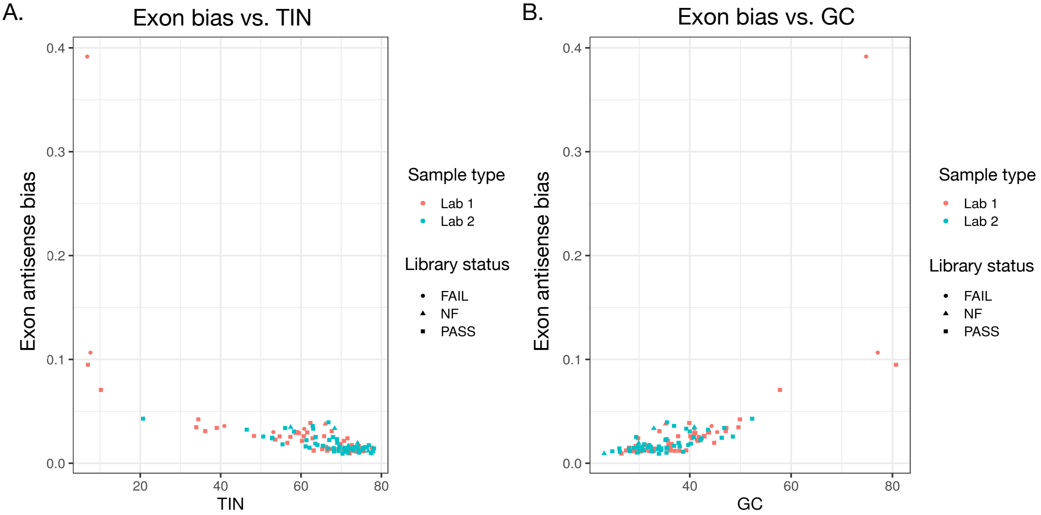

Supplementary Figure 2: Exon bias vs. TIN (A) and GC contents (B). Exon bias correlates negatively with TIN and positively with GC contents.
