# Supplementary figures and images for: Large scale, robust, and accurate whole transcriptome profiling from clinical formalin-fixed paraffin-embedded samples
Source: Sci Rep. 2020 Oct 19;10:17597. doi: 10.1038/s41598-020-74483-1 (PMC7572424; doi:10.1038/s41598-020-74483-1)

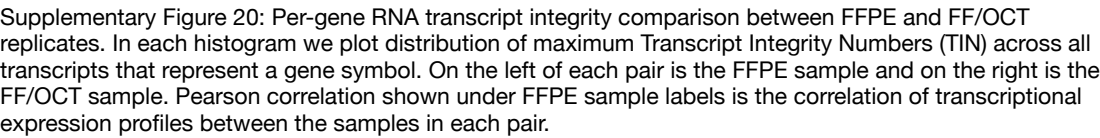

Supplement: Supplementary file 17 — Supplementary Figure 13. [file 41598_2020_74483_MOESM17_ESM.pdf]
