# Supplementary material for: Large scale, robust, and accurate whole transcriptome profiling from clinical formalin-fixed paraffin-embedded samples
Source: Sci Rep. 2020 Oct 19;10:17597. doi: 10.1038/s41598-020-74483-1 (PMC7572424; doi:10.1038/s41598-020-74483-1)
Supplement: Supplementary file 18 — Supplementary Figure 14. [file 41598_2020_74483_MOESM18_ESM.pdf]

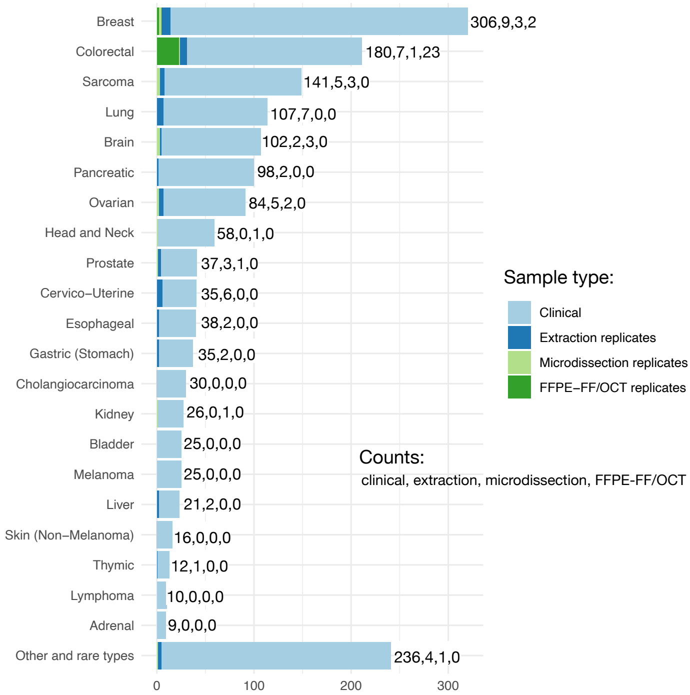

Supplementary Figure 21: Distribution of cancer types within this study's FFPE cohort. The counts are listed by the sample type, separated by comma (clinical, extraction replicates, microdissection replicates, FFPE-FF/OCT replicates). Only those samples with cancer type annotations available are used in these counts. Excluded from this plot are 1,485 unlabeled and research samples.
