# Supplementary material for: Large scale, robust, and accurate whole transcriptome profiling from clinical formalin-fixed paraffin-embedded samples
Source: Sci Rep. 2020 Oct 19;10:17597. doi: 10.1038/s41598-020-74483-1 (PMC7572424; doi:10.1038/s41598-020-74483-1)
Supplement: Supplementary file 19 — Supplementary Figure 15. [file 41598_2020_74483_MOESM19_ESM.pdf]

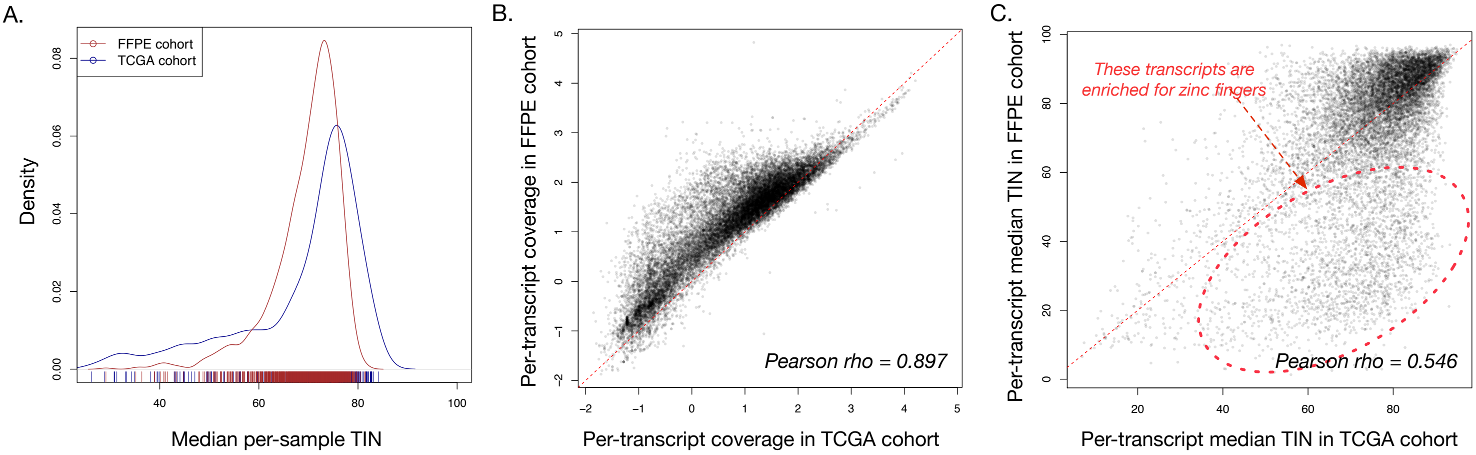

Supplementary Figure 22: Analysis of various sample quality metrics in our study's FFPE cohort vs. TCGA samples. A) Median per-sample TIN as density plots. B) Per-transcript coverage scatterplot shows that our study has better coverage for most transcripts. C) Per-transcript median TIN shows that, while most transcripts show concordance in quality, a subset of transcripts have higher quality in TCGA. These transcripts are significantly enriched in those coding for zinc finger genes.
