# Supplementary material for: Large scale, robust, and accurate whole transcriptome profiling from clinical formalin-fixed paraffin-embedded samples
Source: Sci Rep. 2020 Oct 19;10:17597. doi: 10.1038/s41598-020-74483-1 (PMC7572424; doi:10.1038/s41598-020-74483-1)
Supplement: Supplementary file 20 — Supplementary Figure 16. [file 41598_2020_74483_MOESM20_ESM.pdf]

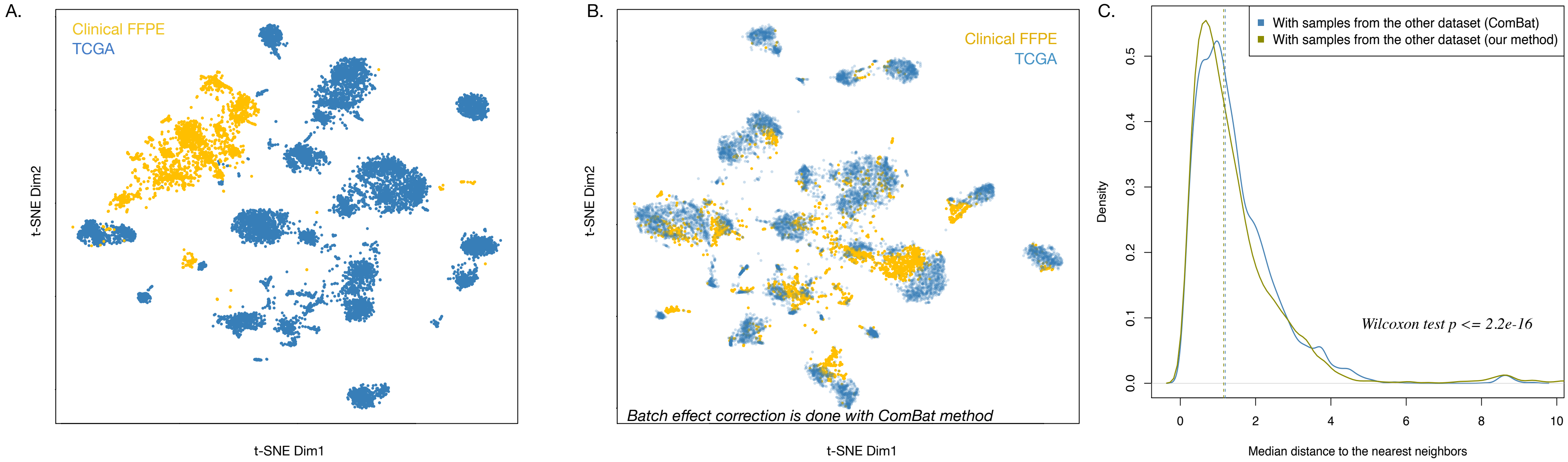

Supplementary Figure 23: Comparison of mapping of TCGA RNA-Seq data into clinical FFPE using ComBat method vs. the method proposed by our study. A) t-SNE projection of the combined data, before mapping TCGA into clinical FFPE, showing which dataset each sample belongs to. B) t-SNE projection of the combined data, after mapping TCGA into clinical FFPE, showing which dataset each sample belongs to. C) Comparison of distance between clinical FFPE and TCGA datasets after TCGA dataset is mapped/projected into clinical FFPE dataset using ComBat method and using our proposed method (see Supplemental Methods). We measured the amount of "scatter", or clumpiness, of the two datasets by computing, for each point in part B of this figure as well as part D of Figure 5, the Euclidean distance between that point and top 5 nearest points in the 2-D space from the other dataset. For each point we computed the median distance between these neighbors. The density plots represent the median distances. The dashed lines indicate median values of each distribution. This plot shows that our method produces more "mixing" (smaller distance) between the datasets.
