# Supplementary material for: Large scale, robust, and accurate whole transcriptome profiling from clinical formalin-fixed paraffin-embedded samples
Source: Sci Rep. 2020 Oct 19;10:17597. doi: 10.1038/s41598-020-74483-1 (PMC7572424; doi:10.1038/s41598-020-74483-1)
Supplement: Supplementary file 21 — Supplementary Figure 17. [file 41598_2020_74483_MOESM21_ESM.pdf]

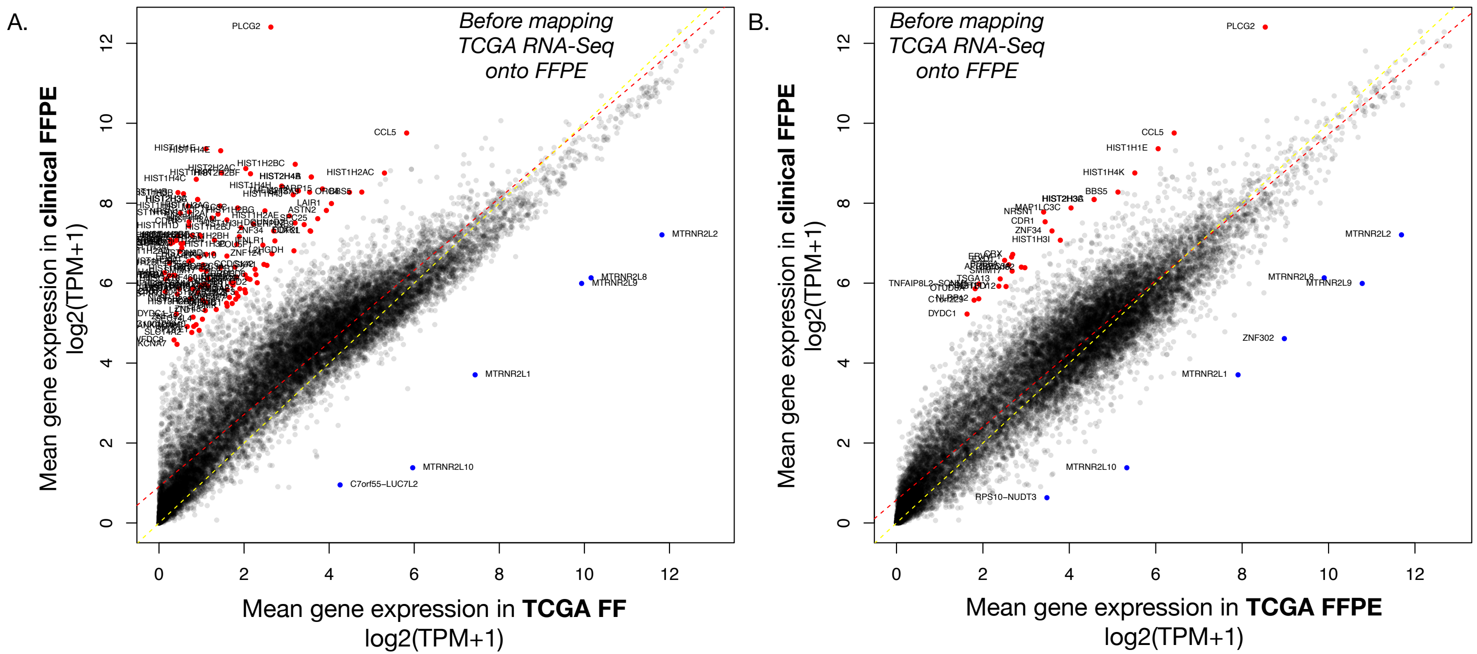

Supplementary Figure 24: Comparison of per-gene mean expression between this study's FFPE cohort and TCGA samples before mapping of TCGA into FFPE RNA-Seq. A) Comparison against TCGA FF samples. B) Comparison against TCGA FFPE samples.
