# Supplementary material for: Large scale, robust, and accurate whole transcriptome profiling from clinical formalin-fixed paraffin-embedded samples
Source: Sci Rep. 2020 Oct 19;10:17597. doi: 10.1038/s41598-020-74483-1 (PMC7572424; doi:10.1038/s41598-020-74483-1)
Supplement: Supplementary file 22 — Supplementary Figure 18. [file 41598_2020_74483_MOESM22_ESM.pdf]

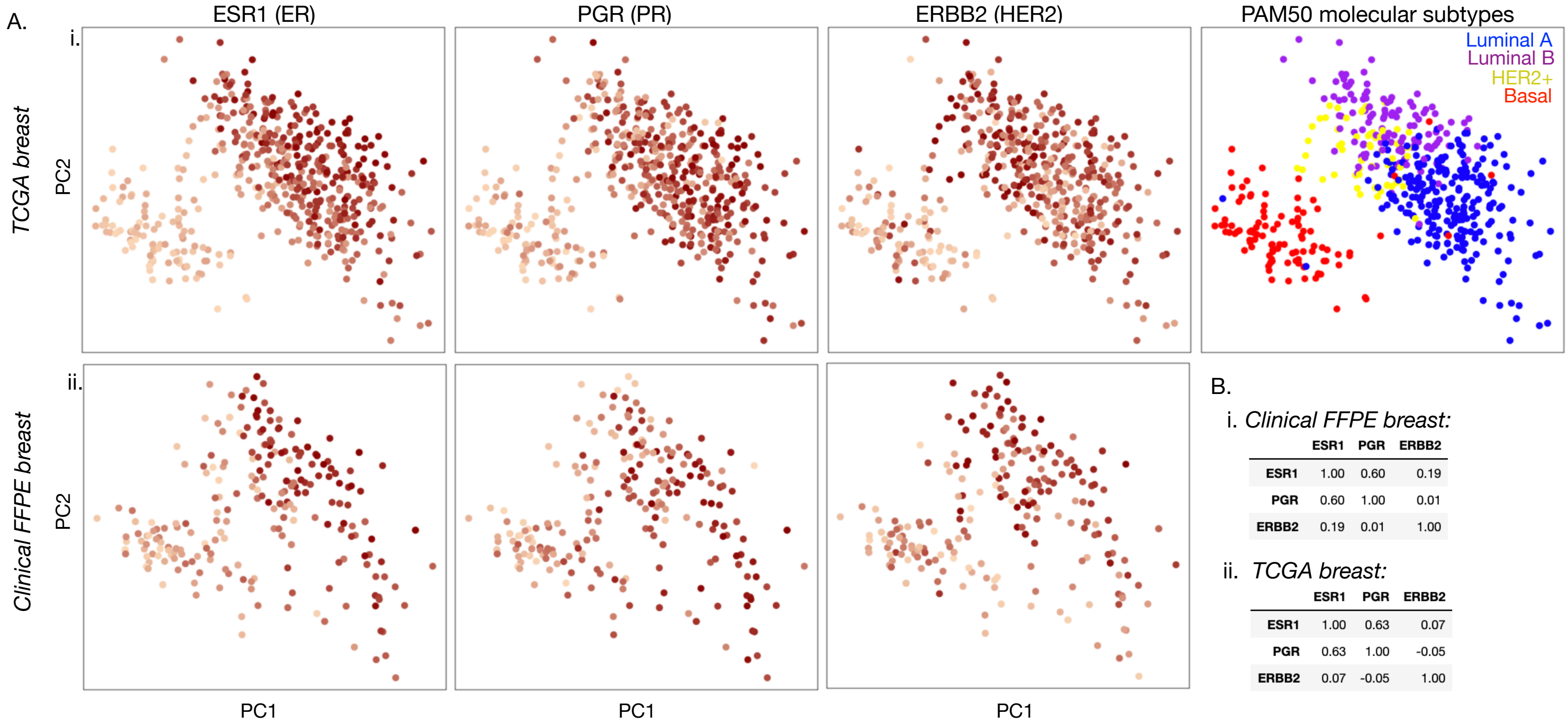

i. *Clinical FFPE breast:*

	ESR1	PGR	ERBB2
**ESR1**	1.00	0.60	0.19
**PGR**	0.60	1.00	0.01
**ERBB2**	0.19	0.01	1.00

ii. *TCGA breast:*

	ESR1	PGR	ERBB2
**ESR1**	1.00	0.63	0.07
**PGR**	0.63	1.00	-0.05
**ERBB2**	0.07	-0.05	1.00

Supplementary Figure 25: Predictive value of ER, PR, and HER2 in clinical diagnostics of breast cancer samples, as compared between the TCGA FF and clinical FFPE cohorts. A) Principal component analysis (PCA) of TCGA (i) and FFPE (ii) breast samples with samples colored by expression levels of ER, PR, and HER2, with brighter color indicating higher expression. For TCGA cohort we include a PCA plot with samples colored by their published PAM50 subtypes. B) Correlation matrices of individual biomarkers within the FFPE (i) and TCGA (ii) cohorts.
