# Supplementary material for: Large scale, robust, and accurate whole transcriptome profiling from clinical formalin-fixed paraffin-embedded samples
Source: Sci Rep. 2020 Oct 19;10:17597. doi: 10.1038/s41598-020-74483-1 (PMC7572424; doi:10.1038/s41598-020-74483-1)
Supplement: Supplementary file 24 — Supplementary Figure 20. [file 41598_2020_74483_MOESM24_ESM.pdf]

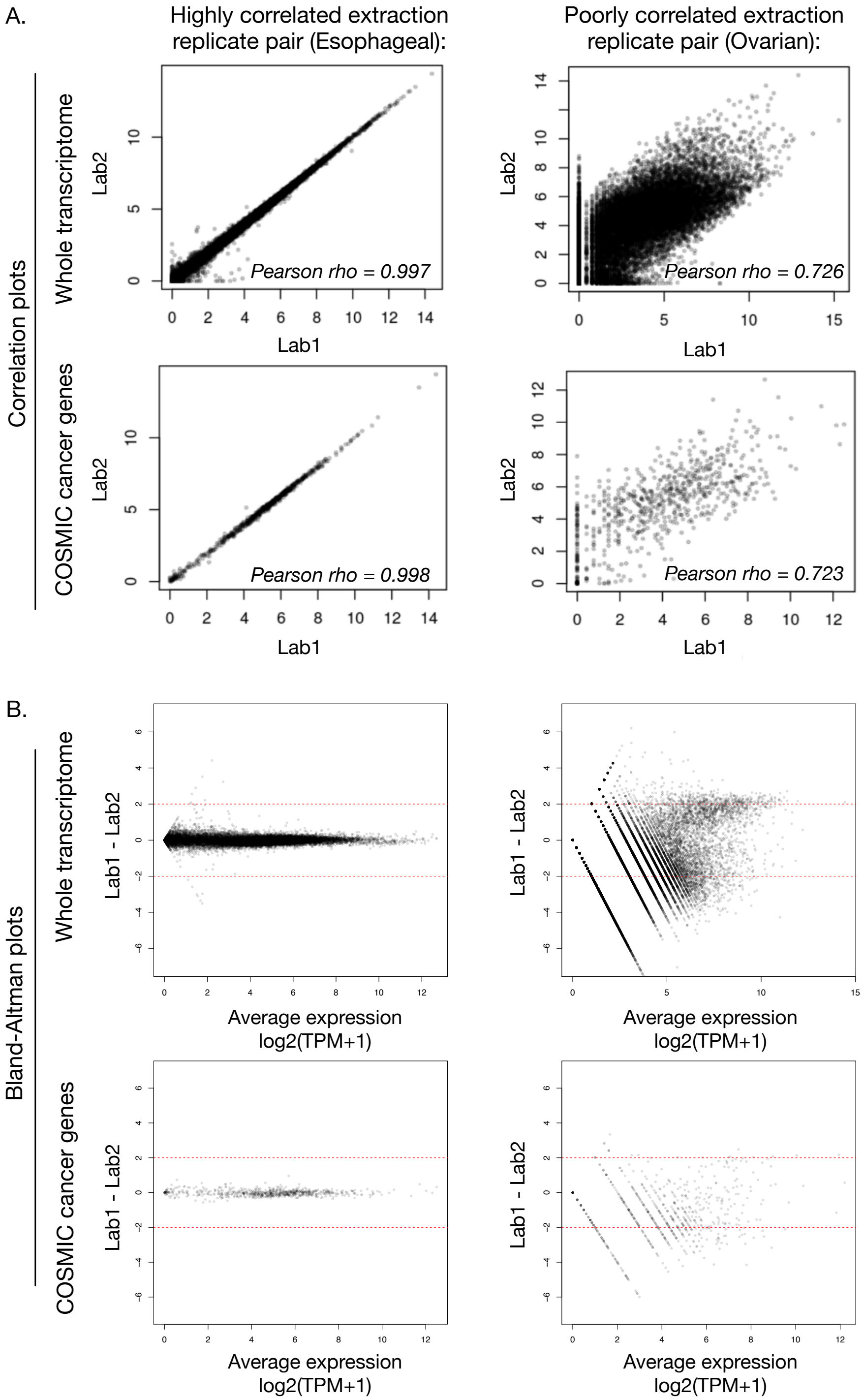

Supplementary Figure 27: Expression comparison of extraction replicates. There are too many samples for a single figure, so in the interest of space we show two examples of highly (on the left) and poorly (on the right) correlated replicates. Two columns show representative pairs with high (left) and low (right) correlations. A) Correlation plots using different gene sets: whole transcriptome (top) and COSMIC cancer genes (bottom). B) Bland–Altman plots using different gene sets: whole transcriptome (top) and COSMIC cancer genes (bottom).
