# Supplementary material for: Large scale, robust, and accurate whole transcriptome profiling from clinical formalin-fixed paraffin-embedded samples
Source: Sci Rep. 2020 Oct 19;10:17597. doi: 10.1038/s41598-020-74483-1 (PMC7572424; doi:10.1038/s41598-020-74483-1)
Supplement: Supplementary file 27 — Supplementary Figure 23. [file 41598_2020_74483_MOESM27_ESM.pdf]

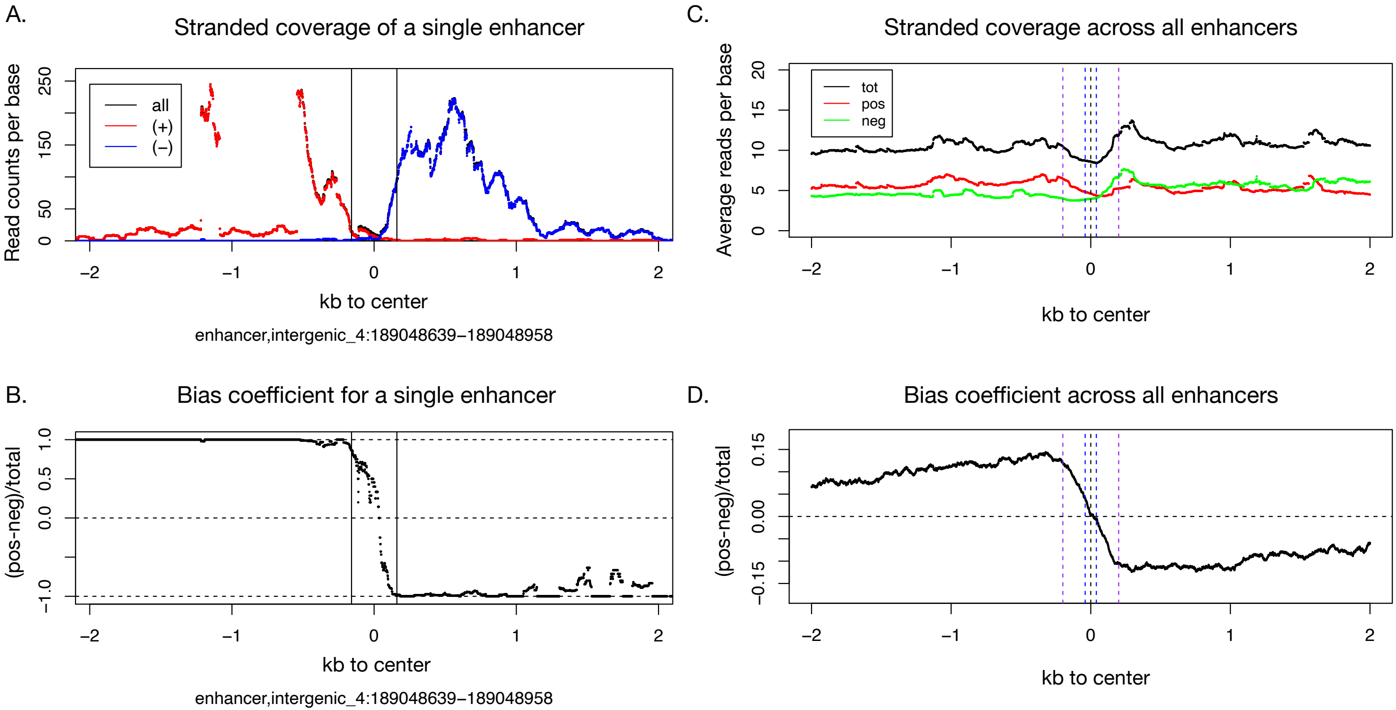

Supplementary Figure 3: Enhancer RNA analysis. A) An example of stranded coverage for a single enhancer. X-axis shows +/- 2kb of the start of the enhancer start site. Blue line shows that coverage of the negative strand and uptake in coverage at the start of enhancer site. B) Bias coefficient plot for the same enhancer as in part A. Bias coefficient describes the fraction of all reads being from positive strand at a given position. C) Stranded coverage summarized across all enhancers of length of at least 80 bases. Green line shows average reads on the negative strand at a given site and shows an increase in negative strandedness at the enhancers' sites. D) Bias coefficient aggregated across all enhancers of length of at least 80 bases.
