# Supplementary material for: Large scale, robust, and accurate whole transcriptome profiling from clinical formalin-fixed paraffin-embedded samples
Source: Sci Rep. 2020 Oct 19;10:17597. doi: 10.1038/s41598-020-74483-1 (PMC7572424; doi:10.1038/s41598-020-74483-1)
Supplement: Supplementary file 28 — Supplementary Figure 24. [file 41598_2020_74483_MOESM28_ESM.pdf]

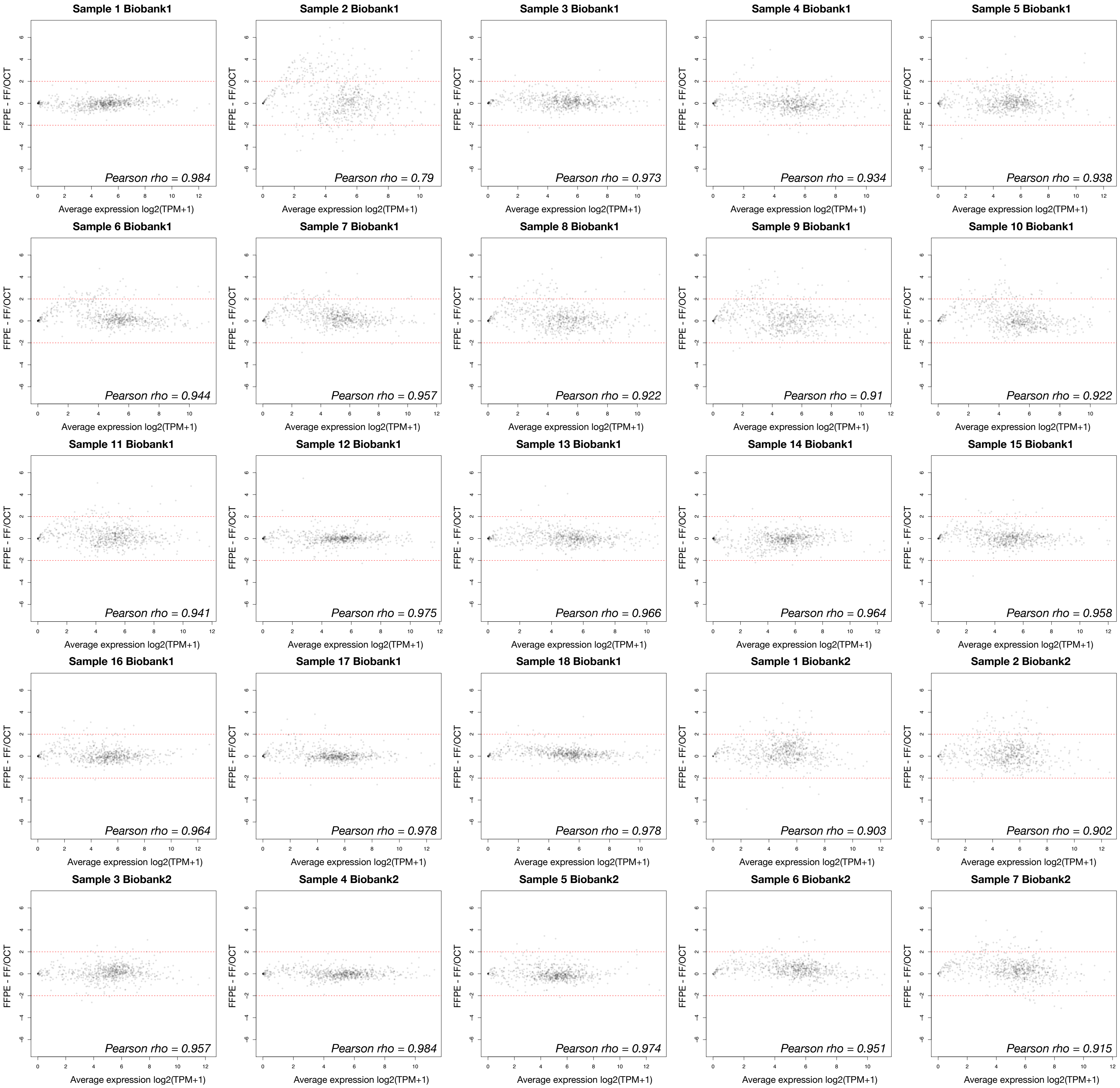

Supplementary Figure 30: Bland–Altman plots using COSMIC cancer genes in log2(rescaled TPM + 1) space of FFPE vs. FF/OCT replicates. All pairs in dataset 1 are colorectal tumor type. Pairs 1-5 in dataset 2 are colon tumor type, while pairs 6 and 7 are breast tumor type. Red line indicates y = x slope. Yellow line indicates linear regression line.
