# Supplementary material for: Large scale, robust, and accurate whole transcriptome profiling from clinical formalin-fixed paraffin-embedded samples
Source: Sci Rep. 2020 Oct 19;10:17597. doi: 10.1038/s41598-020-74483-1 (PMC7572424; doi:10.1038/s41598-020-74483-1)
Supplement: Supplementary file 29 — Supplementary Figure 25. [file 41598_2020_74483_MOESM29_ESM.pdf]

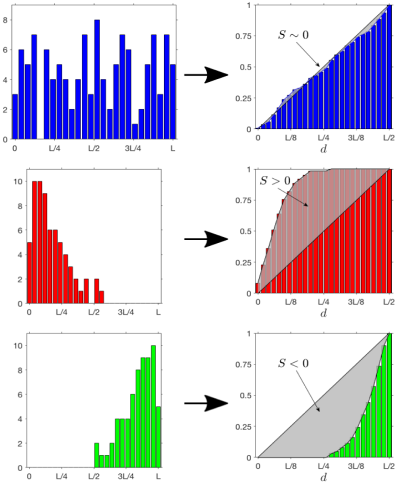

Supplementary Figure 31: Graphical explanation cumulative sums used for the TB score. In the top panel a uniform coverage produces a linear cumulative distribution s_i and thus the integral S is close to zero. In the case of a read coverage biased to the left end of the transcript (middle panel) the cumulative function s_i grows faster than linearly producing a positive value of S and if the coverage is biased to the right end of the transcript (lower panel) then s_i would grow slower than linearly and S have a negative value.
