# Supplementary material for: Large scale, robust, and accurate whole transcriptome profiling from clinical formalin-fixed paraffin-embedded samples
Source: Sci Rep. 2020 Oct 19;10:17597. doi: 10.1038/s41598-020-74483-1 (PMC7572424; doi:10.1038/s41598-020-74483-1)
Supplement: Supplementary file 30 — Supplementary Figure 26. [file 41598_2020_74483_MOESM30_ESM.pdf]

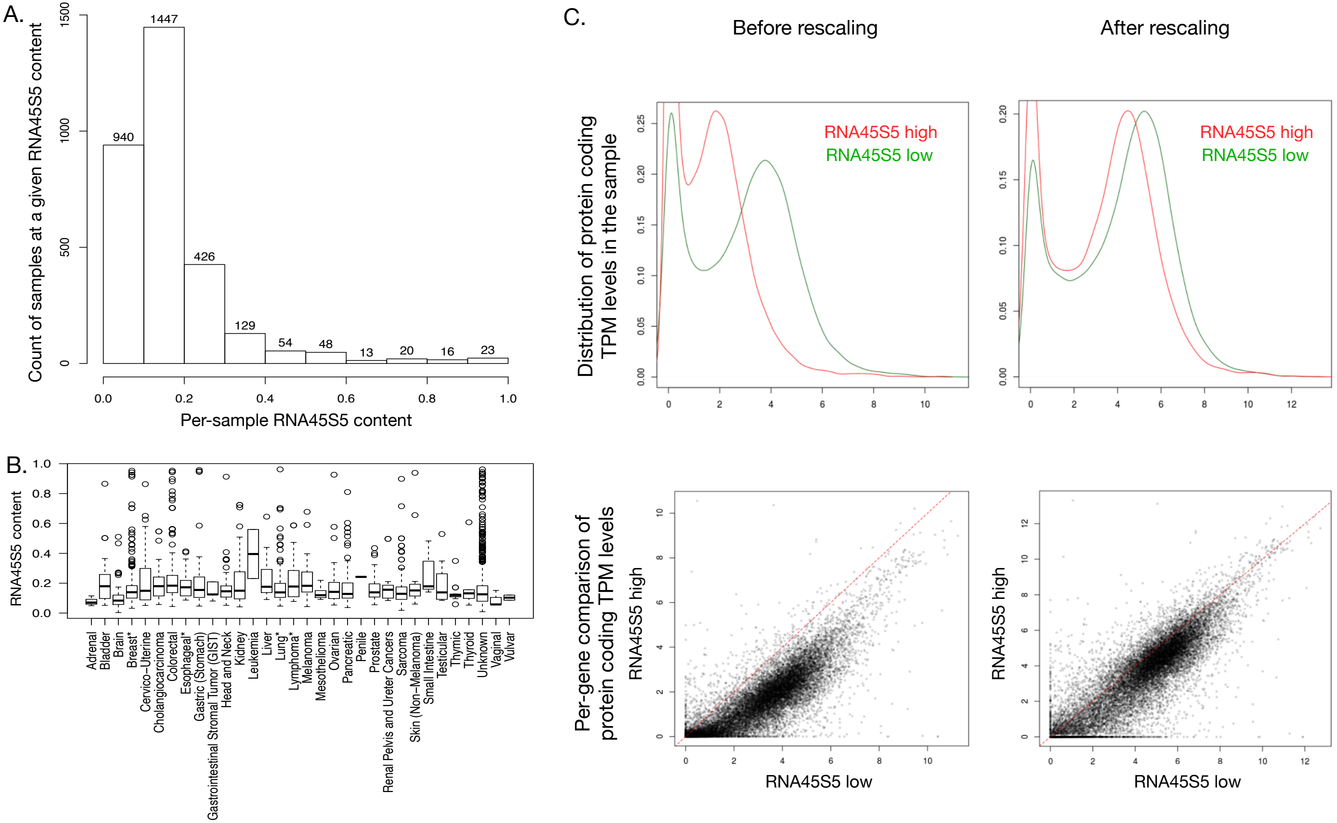

Supplementary Figure 32: Analysis of ribo-depletion quality across the clinical and research FFPE cohort. A) Fraction of RNA45S5 expected counts was computed for each sample as a proxy for amount of ribosomal RNA remaining in the sample after ribo-deplete process. Distribution of these fractions is presented as a histogram. B) RNA45S5 fraction per each cancer type. C) Two breast cancer samples are presented. The first sample has the lowest fraction of RNA45S5 among all breast tumor samples (RNA45S5 low). The second sample has the highest fraction of RNA45S5 among all breast tumor samples (RNA45S5 high). We show that applying per-sample rescaling helps reduce variability driven by technical artifacts and make expression levels more interpretable across biologically similar samples.
