# Supplementary material for: Large scale, robust, and accurate whole transcriptome profiling from clinical formalin-fixed paraffin-embedded samples
Source: Sci Rep. 2020 Oct 19;10:17597. doi: 10.1038/s41598-020-74483-1 (PMC7572424; doi:10.1038/s41598-020-74483-1)
Supplement: Supplementary file 32 — Supplementary Figure 28. [file 41598_2020_74483_MOESM32_ESM.pdf]

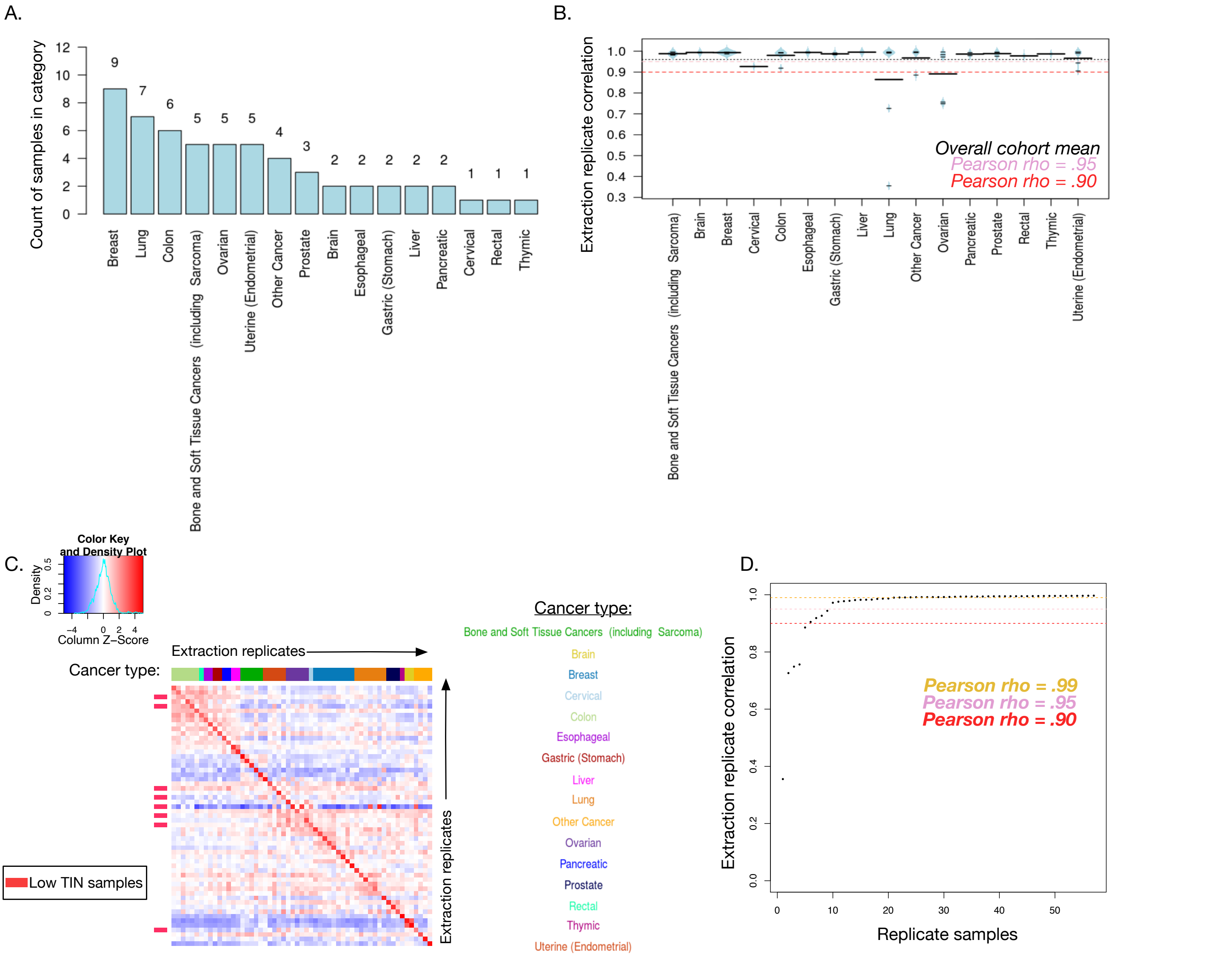

Supplementary Figure 4: Analysis of extraction replicates. A) Distribution of cancer types within extraction replicates cohort. B) Distribution of correlations between extraction replicates by tumor type. C) Correlation heatmap of extraction replicates sorted by cancer type. Both rows and columns are ordered by the same sample order. D) Extraction replicates ordered by their correlations. Yellow line indicates .99, pink line indicates .95, and red line indicates .9 level.
