# Supplementary material for: Large scale, robust, and accurate whole transcriptome profiling from clinical formalin-fixed paraffin-embedded samples
Source: Sci Rep. 2020 Oct 19;10:17597. doi: 10.1038/s41598-020-74483-1 (PMC7572424; doi:10.1038/s41598-020-74483-1)
Supplement: Supplementary file 33 — Supplementary Figure 29. [file 41598_2020_74483_MOESM33_ESM.pdf]

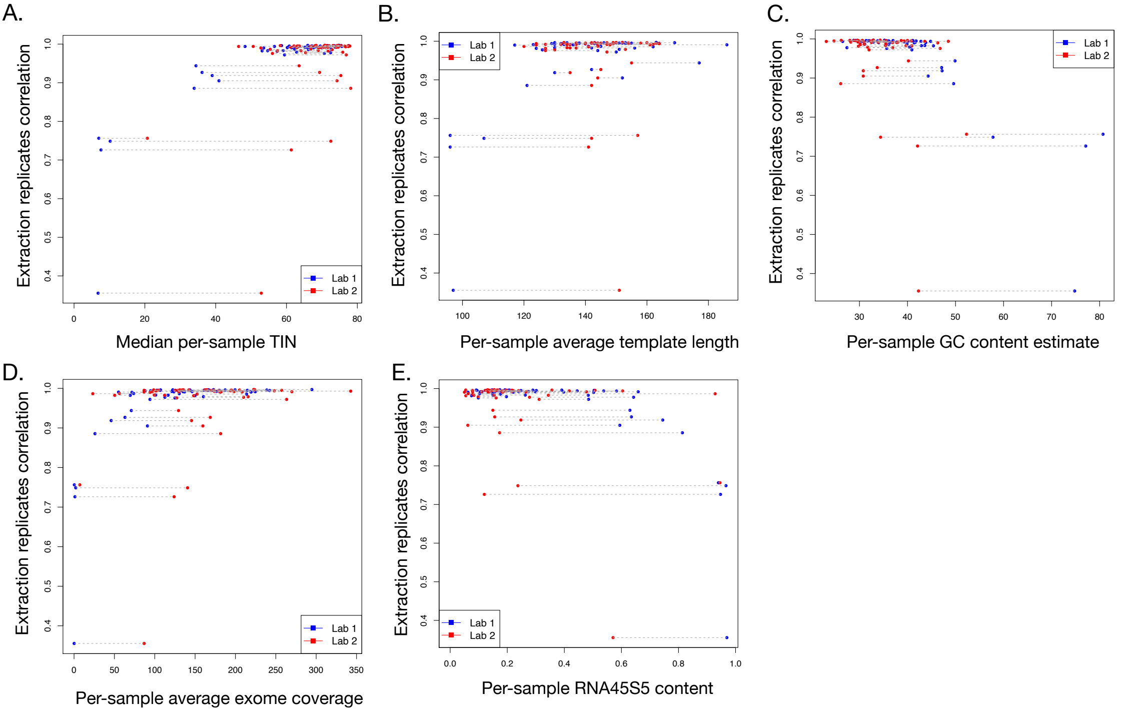

Supplementary Figure 5: Comparison of replicate correlations and various sample quality metrics in direct extraction replicates. For each replicate pair we show a given quality metric for both replicates, on the y-axis we show correlation between these pairs; each replicate pair is connected by a dashed line. A) GC contents. B) Average exome coverage. C) Median per-sample TIN. D) Average template length. E) rRNA depletion quality.
