# Supplementary material for: Large scale, robust, and accurate whole transcriptome profiling from clinical formalin-fixed paraffin-embedded samples
Source: Sci Rep. 2020 Oct 19;10:17597. doi: 10.1038/s41598-020-74483-1 (PMC7572424; doi:10.1038/s41598-020-74483-1)
Supplement: Supplementary file 34 — Supplementary Figure 30. [file 41598_2020_74483_MOESM34_ESM.pdf]

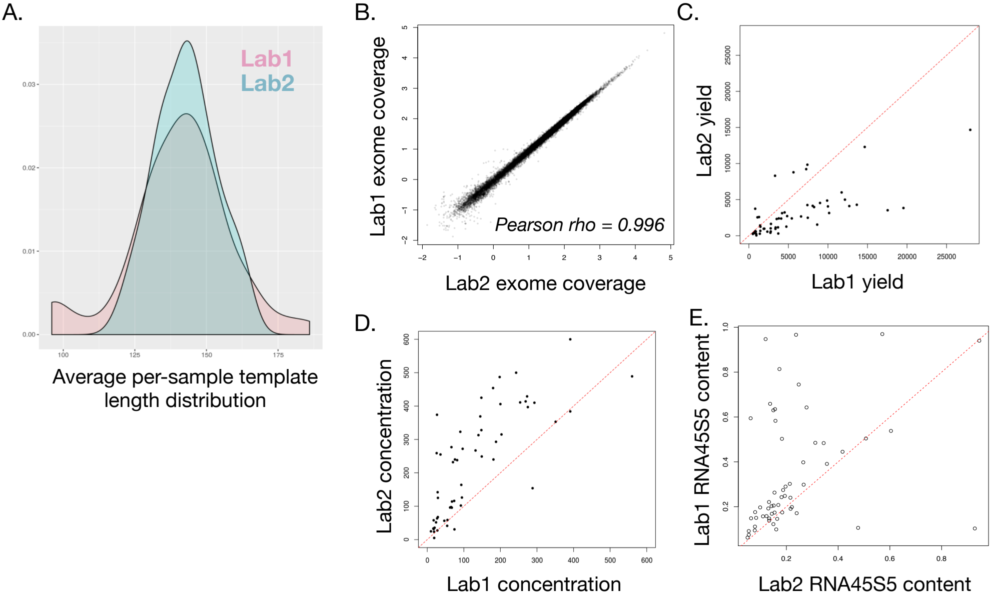

Supplementary Figure 6: Comparison of sample quality metrics in extraction replicates. A) Average template length comparison shows similar results for both labs. B) Average exome coverage is highly correlated between two labs. C-D) Lab2 had higher concentration but lower yield. E) rRNA depletion was better at Lab2.
