# Supplementary material for: Large scale, robust, and accurate whole transcriptome profiling from clinical formalin-fixed paraffin-embedded samples
Source: Sci Rep. 2020 Oct 19;10:17597. doi: 10.1038/s41598-020-74483-1 (PMC7572424; doi:10.1038/s41598-020-74483-1)
Supplement: Supplementary file 35 — Supplementary Figure 31. [file 41598_2020_74483_MOESM35_ESM.pdf]

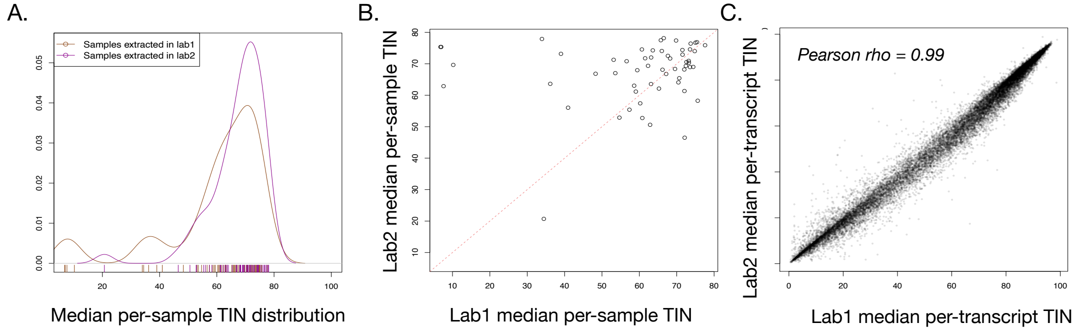

Supplementary Figure 7: Comparison of TIN in extraction replicates. A) Median per-sample TIN is slightly higher in Lab2. B) Median per-sample TIN tends to be higher in Lab2 samples. C) Median per-transcript TIN is highly correlated between two labs.
