# Supplementary material for: Large scale, robust, and accurate whole transcriptome profiling from clinical formalin-fixed paraffin-embedded samples
Source: Sci Rep. 2020 Oct 19;10:17597. doi: 10.1038/s41598-020-74483-1 (PMC7572424; doi:10.1038/s41598-020-74483-1)
Supplement: Supplementary file 36 — Supplementary Figure 32. [file 41598_2020_74483_MOESM36_ESM.pdf]

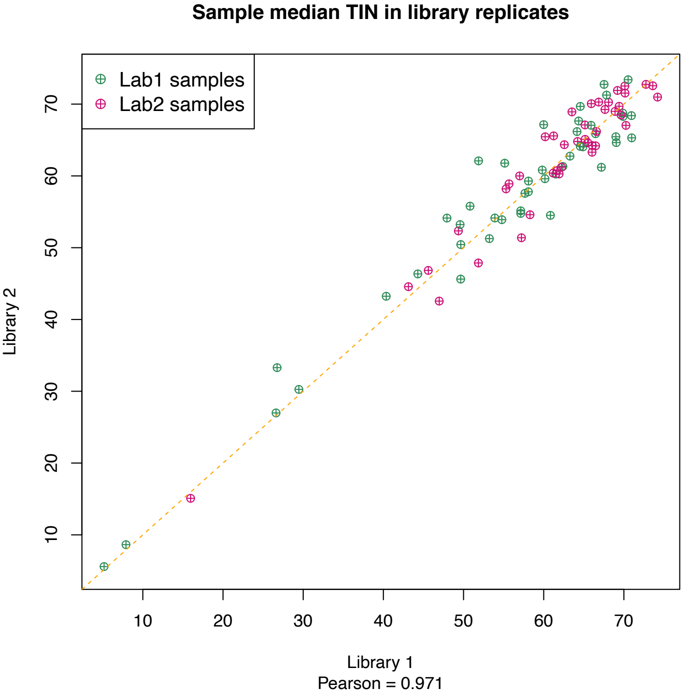

Supplementary Figure 8: Comparison of per-sample median TIN in direct library replicates, showing high reproducibility in sample quality of the same material.
