# Supplementary material for: Large scale, robust, and accurate whole transcriptome profiling from clinical formalin-fixed paraffin-embedded samples
Source: Sci Rep. 2020 Oct 19;10:17597. doi: 10.1038/s41598-020-74483-1 (PMC7572424; doi:10.1038/s41598-020-74483-1)
Supplement: Supplementary file 37 — Supplementary Figure 33. [file 41598_2020_74483_MOESM37_ESM.pdf]

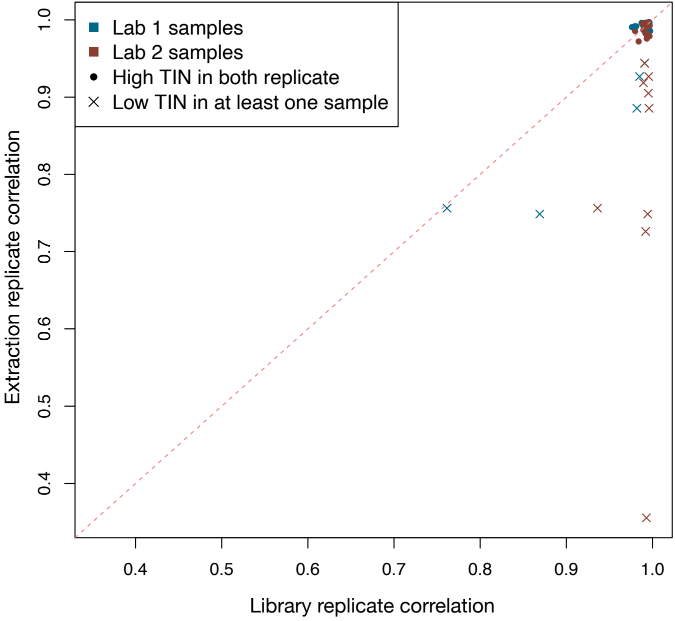

Supplementary Figure 9: Correlations of transcriptional profiles of extraction replicates vs. library replicates. It shows that even when one of the extraction replicates has low quality we can still get highly correlated library replicates from that sample.
